# Reconstructing SNP allele and genotype frequencies from GWAS summary statistics

**DOI:** 10.1038/s41598-022-12185-6

**Published:** 2022-05-17

**Authors:** Zhiyu Yang, Peristera Paschou, Petros Drineas

**Affiliations:** 1 Department of Biological Sciences, Purdue University, West Lafayette, IN USA; 2 Department of Computer Science, Purdue University, West Lafayette, IN USA

**Keywords:** Psychiatric disorders, Genetic association study

## Abstract

The emergence of genome-wide association studies (GWAS) has led to the creation of large repositories of human genetic variation, creating enormous opportunities for genetic research and worldwide collaboration. Methods that are based on GWAS summary statistics seek to leverage such records, overcoming barriers that often exist in individual-level data access while also offering significant computational savings. Such summary-statistics-based applications include GWAS meta-analysis, with and without sample overlap, and case-case GWAS. We compare performance of leading methods for summary-statistics-based genomic analysis and also introduce a novel framework that can unify usual summary-statistics-based implementations via the reconstruction of allelic and genotypic frequencies and counts (ReACt). First, we evaluate ASSET, METAL, and ReACt using both synthetic and real data for GWAS meta-analysis (with and without sample overlap) and find that, while all three methods are comparable in terms of power and error control, ReACt and METAL are faster than ASSET by a factor of at least hundred. We then proceed to evaluate performance of ReACt vs an existing method for case-case GWAS and show comparable performance, with ReACt requiring minimal underlying assumptions and being more user-friendly. Finally, ReACt allows us to evaluate, for the first time, an implementation for calculating polygenic risk score (PRS) for groups of cases and controls based on summary statistics. Our work demonstrates the power of GWAS summary-statistics-based methodologies and the proposed novel method provides a unifying framework and allows further extension of possibilities for researchers seeking to understand the genetics of complex disease.

## Introduction

Genome-wide association studies (GWAS) have emerged as a powerful tool, leading to the identification of thousands of common genetic variants that underlie human complex disorders and traits. They also led to the creation of large repositories of human genetic variation creating enormous opportunities for further analysis. However, sharing and transferring of individual-level genotype data is often restricted due to privacy concerns as well as logistical issues. On the other hand, GWAS summary statistics, typically including information such as odds ratio (OR)/effect size (beta), standard error (SE), *p*-values, and case/control sample sizes for each SNP being analyzed, are often readily accessible^1^. The availability of such alternative sources of information has spurred intense interest into the development of methodologies seeking to leverage such records effectively in order to retrieve as much information as possible. Besides overcoming barriers in individual-level data access, summary-statistics-based methods also offer advantages in computational costs, which do not scale as a function of the number of individuals in the study^2^.

Summary statistics methodologies have been developed to allow a wide array of statistical analyses, including effect size distribution estimation^3,4^; GWAS meta-analysis and fine mapping^5–9^; allele frequency and association statistic imputation^10,11^; heritability and genetic correlation estimation^12–15^; case-case GWAS^16^; and polygenic prediction^17–19^. Many of these methods have to incorporate additional information from publicly available sources, such as linkage disequilibrium (LD) statistics from a reference population^10,12,20^. Most of the existing methodologies analyzing GWAS summary statistics use the summary statistics (OR, SE, *p*-value) from the input “as is”, often via relatively complicated estimation and modeling.

The objective of our work is three-fold. First, we seek to unify GWAS summary-statistics-based analyses (meta-analyses and cc-GWAS) under a common framework, as well as determine whether such frameworks can lead to novel analyses using only summary statistics. Second, we seek to compare existing summary-statistics-based analyses to each other and also our newly introduced method. Third, we present a novel approach to compute statistics that can be used to evaluate the performance of polygenic risk scores *without* accessing individual level genotype data. Our framework leverages a very straightforward observation: summary statistics information can be expressed as a function of case/control allele frequencies for each SNP. This allows us to recover case/control allele frequencies from summary statistics by solving a non-linear system of equations. Additionally, if one assumes that the SNPs satisfy Hardy-Weinberg Equilibrium (HWE) (a common and minimal assumption in all standard GWAS), the allele frequencies can be used to infer genotype counts. These simple observations allow us to use information from case-control GWAS summary statistics to develop a simple, user-friendly alternative to summary-statistics-based methods for fixed effect meta-analysis and cc-GWAS. Furthermore, we are able to compute group-wise polygenic risk score (PRS) from summary statistics of both a base and a target population. We note that even though there have been summary statistics based methods estimating the variance explained by SNPs using results from existing PRS associations^21,22^, to the best of our knowledge, no existing method could return reliable estimates of PRS without any access to individual-level data in the validation cohort prior to our work.

Here, we describe the mathematical foundations of our framework and its applications to fixed effect meta-analysis, cc-GWAS, and group-wise PRS estimation. We compare the performance of existing methods as well as our novel method using both simulated and real data. Our methods are implemented in the software package Reconstructing Allelic Count (ReACt).

## Results

### Mathematical foundations

Our framework is motivated by the fact that the summary test statistics from publicly available GWAS can be expressed as a function of allele counts of the effect and the non-effective allele in cases and controls; as a result, the allele counts can be exactly recovered by solving a system of non-linear equations. Interestingly, this rather straight-forward observation has not received much attention in prior work. Additionally, assuming that SNPs included in GWAS studies are in Hardy-Weinberg Equilibrium (HWE), we can also reconstruct the structure of the genotype vectors for publicly available GWAS studies from just summary statistics. We can leverage this information in multiple applications, including: *(i)* the computation of the joint effect of a SNP in a meta-analysis involving multiple studies; *(ii)* to obtain the mean polygenic risk score of cases and controls in a population; and *(iii)* to investigate the genetic differences between traits using a case-case GWAS. All of these can be done using only summary statistics, which circumvents the hassle of individual level data sharing and, as an added bonus, considerably reduces the necessary computational time. We start by introducing some notation that will be useful in this section. Let *a* and *u* represent effective and non-effective allele counts respectively; let superscripts $^{\text {cse}}$ and $^{\text {cnt}}$ represent cases and controls respectively; and let *OR*, *SE*, and *N* be the odds ratio, standard error (of *log*(*OR*), as presented in most of the GWAS summary statistics), and sample sizes obtained from the summary statistics. Thus, for SNP *i*, $u_{i}^{\text {cnt}}$ represents the count of the non-effective allele in controls for SNP *i*; similarly, $a_{i}^{\text {cse}}$ represents the count of the effective allele in cases for SNP *i*; $N^{\text {cse}}$ represents the number of cases, etc. We now note that the allelic effect of SNP *i* in case-control GWAS summary statistics can be expressed as follows:

$$\begin{aligned} OR_{i}= & {} \frac{a_{i}^{\text {cse}} \cdot u_{i}^{\text {cnt}}}{a_{i}^{\text {cnt}} \cdot u_{i}^{\text {cse}}},\\ SE_{i}= & {} \sqrt{\frac{1}{a_{i}^{\text {cse}}} + \frac{1}{u_{i}^{\text {cse}}} + \frac{1}{a_{i}^{\text {cnt}}} + \frac{1}{u_{i}^{\text {cnt}}}} \end{aligned}$$

Additionally, sample sizes can be expressed as:

$$\begin{aligned} 2N^{\text {cse}}= & {} a_{i}^{\text {cse}} + u_{i}^{\text {cse}},\quad \text {and} \\ 2N^{\text {cnt}}= & {} a_{i}^{\text {cnt}} + u_{i}^{\text {cnt}}. \end{aligned}$$

Therefore, solving the system of the above four non-linear equations allows us to recover the allelic counts of SNP *i* for effective and non-effective alleles in cases and controls, by solving for the four unknowns $a_{i}^{\text {cse}}$, $a_{i}^{\text {cnt}}$, $u_{i}^{\text {cse}}$, and $u_{i}^{\text {cnt}}$. Using these counts, we can trivially obtain allele frequencies in case and control groups and, importantly, by further assuming that the SNPs strictly follow HWE, we can even compute the genotypic counts for each genotype from these frequencies. Note that this approach applies to GWAS reporting *OR* and *SE* statistics for each SNP, or perhaps other statistics that can be used to compute *OR* and *SE*; it may not be applicable to GWAS reporting other types of summary statistics. Furthermore, these frequencies will be different from those observed from individual level data due to model covariates; the recovered frequencies correspond to the allele counts after corrections have been applied. See Section 4.1 and 5.2 in supplementary text for details.

### Fixed effect meta-analysis

#### Our approach

Armed with allelic and genotypic counts, we can provide a new perspective on fixed-effect GWAS meta-analysis. Instead of the conventional inverse-variance weighted meta-analysis, we can now compute the joint effect of a SNP in a meta-analysis using multiple studies by combining the reconstructed allele and genotype counts from each study and run a *complete* logistic regression on each SNP. Thus, we can essentially proceed with the analysis in exactly the same way as standard GWAS (see “Fixed-effect meta-analysis” section for details).

As mentioned in “Mathematical foundations” section we can obtain genotypic counts for any SNP over cases and controls from GWAS summary statistics. Then, combining these counts for all available input studies, along with the trait status, we can carry out a logistic regression for this SNP as follows^23^:

$$\begin{aligned} {{\mathsf {Pr}}}({\mathbf {y}}_j = 1 | {\mathbf {g}}_j,{\mathbf {s}}_j) = S(\beta _0 + \beta _1 {\mathbf {g}}_j + \beta _2 {\mathbf {s}}_j). \end{aligned}$$

In the above ${\mathbf {y}}_j$ denotes the binary trait for the *j*th individual, ${\mathbf {g}}_j$ denotes the respective genotype, and $S(\cdot )$ stands for the standard sigmoid function used in logistic regression. Solving for the coefficients $\beta _0$, $\beta _1$, and $\beta _2$ we get the overall SNP effect from the meta-analysis. In order to take into account between-study stratification, we introduce an additional variable ${\mathbf {s}}_j$ as a covariate, using the overall allele frequencies of each study to estimate it (see “Fixed-effect meta-analysis” section for details).

#### Fixed effect meta-analysis: performance evaluation

**Figure 1:**
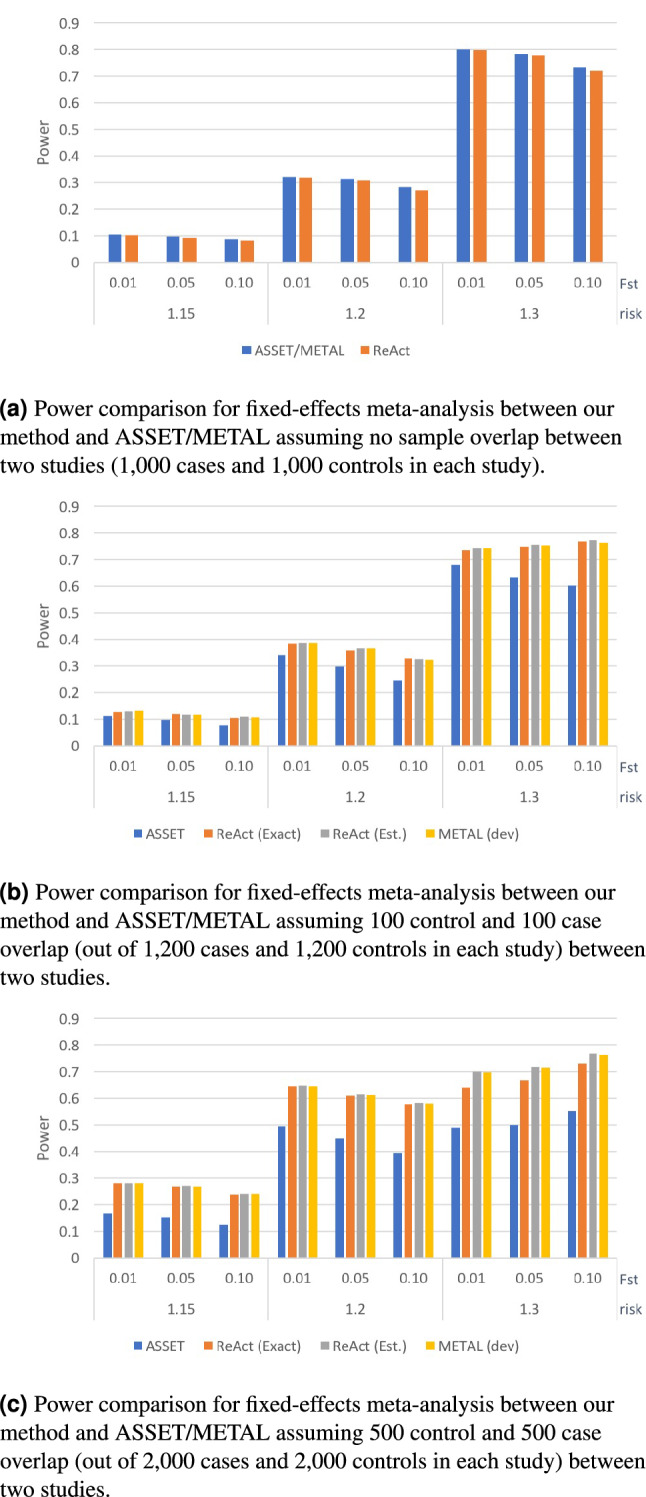
Power of fixed-effect meta-analysis with two input studies under different conditions. We compare the power of our method vs. ASSET/METAL for a significance threshold $p < 5 \cdot 10^{-5}$. METAL dev refers to the latest release in GitHub^28^. Two variants of ReACt are tested: Exact and Est, indicating whether the sample overlap was *exactly* known as part of the input or whether it was *estimated* from the *Z*-scores^28^, respectively. Sample overlap indicates the number of cases and controls that were shared between two input studies, ie., a sample overlap equal to 100 means that there are 100 cases and 100 controls shared between two input studies. Total sample sizes for each input study, including the shared samples, are equal to 2000 when the sample overlap is equal to zero; 2400 when the sample overlap is equal to 100; and 4000 when the sample overlap is equal to 500. In each case, the sample is equally split to cases and controls.

**Figure 2:**
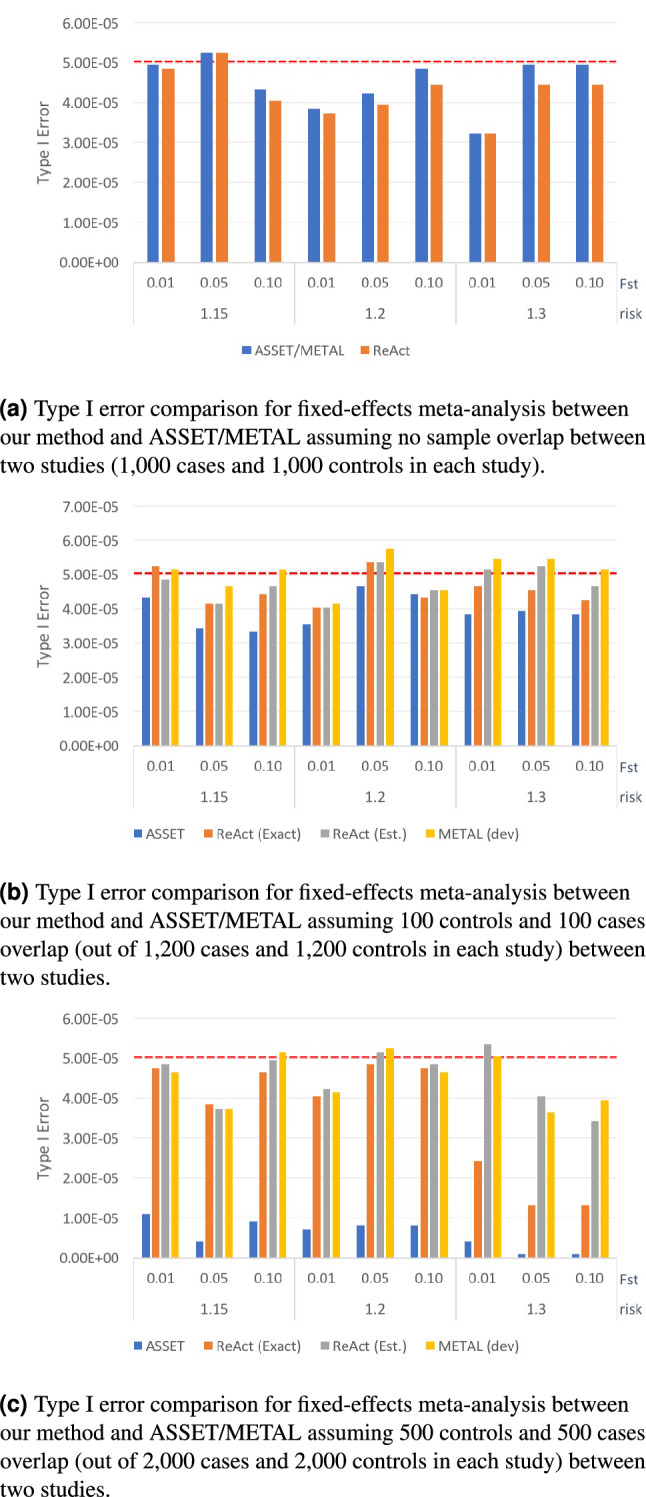
Type I error rate of fixed-effect meta-analysis with two input studies under different conditions. We compared the type I error rate of our method vs. ASSET/METAL for a significance threshold $p < 5 \cdot 10^{-5}$. METAL dev refers to the latest release in GitHub^28^. Two variants of ReACt are tested: Exact and Est, indicating whether the sample overlap was *exactly* known as part of the input or whether it was *estimated* from the *Z*-scores^28^, respectively. Sample overlap indicates the number of cases and controls that were shared between two input studies, ie., a sample overlap equal to 100 means that there are 100 cases and 100 controls shared between two input studies. Total sample sizes for each input study, including the shared samples, are equal to 2000 when the sample overlap is equal to zero; 2400 when the sample overlap is equal to 100; and 4000 when the sample overlap is equal to 500. In each case, the sample is equally split to cases and controls.

First, we tested the performance of two leading methods used for fixed-effect meta-analysis (namely METAL^24^ and ASSET^25^) as well as ReACt on synthetic data under various conditions. The simulation was carried out using the Balding-Nichols model^26^, assuming a minor allele frequency of 0.3. For each setting, we predefined the risk for effective alleles of the causal SNPs by setting $r = 1.15/1.2/1.3$ as well as the level of population stratification between cohorts included in the meta-analysis setting $F_{st} = 0.01/0.05/0.1$. Apart from meta-analyzing mutually exclusive datasets, we also tested the performance of all three methods under different extents of sample overlap between the input studies: When generating input summary statistics, we evaluated scenarios where the input studies shared $N_{\texttt {shr}}$ cases and $N_{\texttt {shr}}$ controls, with the value of $N_{\texttt {shr}}$ set to zero, 100, and 500 (see “Data” section for details). ASSET corrects for known sample overlap through introducing correlations between summary statistics derived from overlapping and input sample sizes^25,27^. Since the latest stable release of METAL does not include an implementation for sample overlap correction, we used the GitHub version of METAL from^28^. ReACt allows the user to provide the overlapping sample sizes as an input parameter (ReACt(Exact) in Figs. 1, 2). Furthermore, same as METAL, it allows the estimation of unknown sample overlap via *Z*-scores in input GWAS summary statistics from^28^ (ReACt(Est.) in Figs. 1, 2). We compared power and type I error rates of all three tested approaches.

The performance comparison on the meta-analysis of two studies with even case/control sample sizes is plotted in Figs. 1, 2 and Table S3. Performance on meta-analyzing two studies with uneven sample sizes (Table S2 in supplementary text) as well as meta-analyzing multiple studies (Table S4 in supplementary text) are also tested. Results on synthetic data indicated that all three methods have comparable performance namely

$$\begin{aligned} \left| {{\mathrm {Power}}}_\mathbf{ReACt } - {{\mathrm {Power}}}_\mathbf{ASSET/METAL }\right| \le 0.012, \end{aligned}$$

when there is no sample overlap. In scenarios where there were samples shared across input studies, METAL and ReACt (regardless of whether the exact size of the sample overlap is known or is estimated) always showed higher power compared to ASSET

$$\begin{aligned}&0.014 \le {{\mathrm {Power}}}_\mathbf{ReACt/METAL } - {{\mathrm {Power}}}_\mathbf{ASSET } \le 0.219\\&\left| {{\mathrm {Power}}}_\mathbf{ReACt } - {{\mathrm {Power}}}_\mathbf{METAL }\right| \le 0.005. \end{aligned}$$

The advantage in power for our method and METAL compared to ASSET was more visible under higher $F_{st}$ values and larger sample overlaps. In terms of type I error rates, we observed that all methods showed good control on the error rates, while ASSET tended to produce more conservative results. Similar observations can also be made when we meta-analyzed multiple studies; see Table S4 in supplementary text for details.

Beyond power and type I error, we also analyzed the running time of the different methods (see Table S1 in supplementary text). METAL and ReACt far outperform ASSET in this regard. It should be noted that our C/C++ implementation of our method in the ReACt software package is comparable (in terms of running time) to METAL and much faster than ASSET, despite the fact that it has not been highly optimized for performance.

To demonstrate the scalability of ReACt beyong the Balding-Nichols model, we also looked at the performance of ReACt on phenotypes simulated using the UK biobank genotypes and the gcta tool^29^ (see “Data” section for details). In our simulation, we set the heritability parameter to 0.4 and the disease prevalence to 0.2. We do note that, theoretically, the performance of ReACt should be invariant to disease heritability or prevalence. For this experiment, all causal SNPs were defined to have effect sizes $OR = 1.2$ and we evaluated the performance of all methods by comparing them with results from GWAS on all samples (see “Evaluation metrics” section for details). See Table 1 for our experimental results. We found all methods having comparable power and type I error. More specifically, the performance of ReACt(Exact) and ASSET showed high similarity and so did the performance of ReACt(Est) and METAL.

**Table 1:** Performance of fixed-effect meta-analysis on simulated data using the gcta model.

Method	No sample overlap$^{\mathrm{a}}$		5000 sample overlap$^{\mathrm{b}}$		10,000 sample overlap$^{\mathrm{c}}$	
	Power	Type I error	Power	Type I error	Power	Type I error
ReACt (Exact)	0.9738	$7.32\times 10^{-5}$	0.8976	$6.43 \times 10^{-4}$	0.8757	$6.81 \times 10^{-4}$
ReACt (Est.)	–	–	0.9120	$8.36 \times 10^{-5}$	0.8794	$7.24 \times 10^{-5}$
METAL/METAL dev	0.9748	$7.55\times 10^{-5}$	0.9111	$8.23\times 10^{-5}$	0.8779	$7.13 \times 10^{-5}$
ASSET	–	–	0.8898	$5.69\times 10^{-5}$	0.8660	$5.89 \times 10^{-5}$

Using the simulated phenotypes for UK biobank samples (50,000 cases and 250,000 controls), we compared the performance of our method vs. ASSET/METAL. We treated genome-wide significant SNPs (*p*-value $< 5 \times 10^{-8}$) as “true signals”, and reported average power and type I error rates on identifying those SNPs under the same genome-wide significance threshold for each method. METAL dev refers to the latest release in GitHub^28^. Two variants of ReACt are tested: Exact and Est, indicating whether the sample overlap was *exactly* known as part of the input or whether it was *estimated*, respectively. Sample overlap indicates the number of cases and controls that were shared between two input studies, i.e., 5000 sample overlap means that 5000 cases and 5000 controls were shared between the two studies when the split was carried out.

^a^With 25,000 cases and 125,000 controls from each subset.

^b^Out of 27,500 cases and 127,500 controls from each subset.

^c^Out of 30,000 cases and 130,000 controls from each subset.

We further tested the performance of all three methods on real genotype and phenotype data using the UK biobank dataset^30^ and analyzing for depressive episode trait. The dataset included a total of 18,368 cases, 312,849 controls, with 640,756 SNPs after quality control (see “Data” section for details). In this experiment, we treated the top 7 SNPs with *p*-value strictly less than $10^{-6}$ from the overall GWAS as “ground truth” and assessed whether various meta-analysis method could pick up these 7 SNPs. Each experiment was carried out over ten iterations: in each iteration, we split the dataset in two equal sized subsets, generated GWAS summary statistics from each of the subsets, and meta-analyzed the resulting summary statistics. We reported average true positive and false positive SNPs counts captured by each method over the ten iterations. Table 2 reports our findings and we note that, perhaps due to the lack of stratification, the differences in performance were not as visible in experiments using the UK biobank samples compared to the Balding-Nichols simulation. A consistent outcome of both experiments was that ReACt(Exact) showed essentially identical performance with ASSET, whereas ReACt(Est) was more comparable with METAL. This should be expected given that both ReACt(Exact) and ASSET require the size of the sample overlap as input, unlike ReACt(Est) and METAL.

**Table 2:** Performance of fixed-effect meta-analysis on real genotype data.

SNP	P	Number of times the SNP had *p*-value $<10^{-5}$ in meta-analysis									
		No sample overlap$^{\mathrm{a}}$		500 sample overlap$^{\mathrm{b}}$				1000 sample overlap$^{\mathrm{c}}$			
		Exact	ASSET/METAL	Exact	Est.	METAL dev	ASSET	Exact	Est.	METAL dev	ASSET
rs60939828	2.77$\cdot 10^{-9}$	10	10	10	10	10	10	10	10	10	10
rs17487484	2.61$\cdot 10^{-8}$	10	10	10	10	10	10	10	10	10	10
rs62100766	1.55$\cdot 10^{-7}$	10	10	9	9	8	9	9	4	4	9
rs4510098	5.34$\cdot 10^{-7}$	10	10	5	5	5	5	5	4	3	5
rs1079232	6.69$\cdot 10^{-7}$	2	2	5	4	3	5	3	2	2	3
rs75056899	7.69$\cdot 10^{-7}$	10	10	3	3	3	3	4	4	4	4
rs12044988	7.75$\cdot 10^{-7}$	10	10	5	1	1	5	6	4	3	6
True positive per iteration		6.2	6.2	4.7	4.2	4	4.7	4.7	3.8	3.6	4.7
False positive per iteration		0.2	0.2	1.4	0.6	0.4	1.5	1.6	0.5	0.7	1.7

We applied our method for fixed-effect meta-analysis to the depressive episode trait (ICD F32 Depressive episode) in UK biobank samples and compared the performance of our method vs. ASSET/METAL. SNPs with *p*-value strictly less than $10^{-6}$ in the primary GWAS summary statistics using all samples were treated as “true signals”. In each iteration of an experiment, we split the dataset evenly into two, generated GWAS summary statistics for each subset, and meta-analyzed the summary statistics using our method and ASSET/METAL. We reported the number of times (out of ten iterations) that a “true signal” got captured using the “significance threshold” $p < 10^{-6}$ by each method under different sample overlap conditions. METAL dev refers to the latest release in GitHub^28^. Two variants of ReACt are tested: Exact and Est, indicating whether the sample overlap was *exactly* known as part of the input or whether it was *estimated*, respectively. Sample overlap indicates the number of cases and controls that were shared between two input studies, ie., 500 sample overlap means that 500 cases **and** 500 controls were shared between the two studies when the split was carried out. The variable *P* in the table indicates the *p*-value of the target SNP in the primary GWAS using all samples. *True positive per iteration* reports the average number of SNPs with *p*-value strictly less than $10^{-6}$ in the primary GWAS that were captured in one iteration; and *False positive per iteration* reports the average number of extra SNPs being captured in one iteration.

^a^With 9184 cases and 156,425 controls from each subset.

^b^Out of 9434 cases and 156,675 controls from each subset.

^c^Out of 9684 cases and 156,925 controls from each subset.

### cc-GWAS

Case-case GWAS (cc-GWAS) based on summary statistics has only very recently been described by Peyrot et al.^31^. No other methods have been proposed so far. ccGWAS can be used to investigate the genetic differences between the patients of two diseases. With some assumptions on SNP effect distributions, Peyrot et al. described the case-case effect as a weighted sum of SNP effects from each input GWAS, where the weights could be derived from SNP-based heritabilities, prevalence, number of independent causal variants for each disease, and their genetic correlation. We observed that the framework of analysis that we introduced above, although only requiring minimal assumptions and nothing else apart from basic information come along with the GWAS summary statistics (*SE*, *OR*/*Beta* and case control sample sizes), could also be used to implement cc-GWAS under the same umbrella. We proceed here to describe this implementation and comparison of the two methods.

#### Our approach

Similar to our proposed approach for meta-analysis of multiple GWAS datasets using summary statistics, we can also carry out cc-GWAS using regression by simply swapping the labels of the phenotypes. Perhaps the biggest challenge in cc-GWAS is the separation of the differential genetic effects from between-study stratification. To circumvent this issue, we leverage the difference of SNP effects in control groups to estimate the extent of stratification (see “cc-GWAS using summary statistics” section for details). Therefore, with a slight modification of the pipeline for meta-analysis of “Fixed-effect meta-analysis” section, we introduce an alternate approach for cc-GWAS using our framework.

The underlying theory is quite straightforward and allows us to estimate the genetic differences between two traits of interest using their GWAS summary statistics. Using the genotypic counts we can proceed with logistic regression using only the cases from the two studies:

$$\begin{aligned} {{\mathsf {Pr}}}({\mathbf {y}}_j^{{\texttt {cse}}} = 1 | {\mathbf {g}}^{{\texttt {cse}}}_j) = S(\beta _0^{{\texttt {cse}}} + \beta _1^{{\texttt {cse}}} {\mathbf {g}}^{{\texttt {cse}}}_j) \end{aligned}$$

In the above, ${\mathbf {y}}_j^{{\texttt {cse}}}$ is the binary indicator variable denoting which trait case *j* carries and ${\mathbf {g}}^{{\texttt {cse}}}_j$ is the genotype of this case. We note that in an additive model, the coefficient $\beta _1^{{\texttt {cse}}}$ that is part of the output of this regression is a combination of both genetic effects and stratification:

$$\begin{aligned} \beta _1^{{\texttt {cse}}} = \beta _g + \beta _s, \end{aligned}$$

where $\beta _g$ and $\beta _s$ are the genetic effect and stratification coefficients. We are only interested in the genetic effect $\beta _g$ and therefore we need to remove $\beta _s$. Towards that end, we estimate $\beta _s$ using the control samples from the input studies; see “cc-GWAS using summary statistics” section for details.

#### CC-GWAS: performance evaluation

We first tested the performance of our methods on synthetic data. Simulated data were again generated under the Balding-Nichols model, with predefined risks for effective allele of the causal SNPs and the extent of the stratification. Inspired by Peyrot et al.^16^ we simulated three types of SNPs: *(i)* trait differential SNPs *(ii)* null SNPs; and *(iii)* stress SNPs (see “Data” section for details). We expect our method to pick up type (i) SNPs and leave the other two. Therefore, in our performance evaluation, we report the power for detecting the type (i) SNPs and type I error rates for picking up type (ii) and (iii) SNPs. Moreover, since we also expect the performance of our method, especially in terms of error control, to vary with sample size, the evaluation was done under different sample sizes in each input study (2000 cases and 2000 controls as well as 5000 cases and 5000 controls). Power and type I error rates for each type of SNP from the simulation model under each setting are shown in Table 3. The method’s performance was evaluated for *p*-values strictly less than $5 \cdot 10^{-5}$. For this threshold, our method showed high power and well-controlled type I errors, especially under for lower values of $F_{st}$. On the other hand, as expected, as stratification increases between two input studies, the power of our method drop and the type I error rates increased for null SNPs. However, as a general trend, we also see a decrease in such error rates when we increase the control sample size. Meanwhile, slightly higher type I error rates for the stress SNPs are observed.

**Table 3:** Performance of cc-GWAS as implemented in ReACt with different sample sizes.

Risk	Fst	2000 cases, 2000 controls			5000 cases, 5000 controls		
		Power	Type I err.$^{\texttt {(ii)}}$	Type I err.$^{\texttt {(iii)}}$	Power	Type I err.$^{\texttt {(ii)}}$	Type I err.$^{\texttt {(iii)}}$
1.15	0.01	3.67$\cdot 10^{-2}$	2.65$\cdot 10^{-5}$	3.16$\cdot 10^{-4}$	3.51$\cdot 10^{-1}$	1.84$\cdot 10^{-5}$	1.87$\cdot 10^{-4}$
	0.05	3.49$\cdot 10^{-2}$	9.80$\cdot 10^{-5}$	5.26$\cdot 10^{-4}$	3.23$\cdot 10^{-1}$	6.33$\cdot 10^{-5}$	3.58$\cdot 10^{-4}$
	0.1	2.81$\cdot 10^{-2}$	2.43$\cdot 10^{-4}$	5.02$\cdot 10^{-4}$	2.85$\cdot 10^{-1}$	1.94$\cdot 10^{-4}$	5.21$\cdot 10^{-4}$
1.2	0.01	1.54$\cdot 10^{-1}$	4.69$\cdot 10^{-5}$	2.47$\cdot 10^{-4}$	7.16$\cdot 10^{-1}$	3.47$\cdot 10^{-5}$	2.03$\cdot 10^{-4}$
	0.05	1.34$\cdot 10^{-1}$	1.04$\cdot 10^{-4}$	5.14$\cdot 10^{-4}$	6.62$\cdot 10^{-1}$	8.57$\cdot 10^{-5}$	3.77$\cdot 10^{-4}$
	0.1	1.23$\cdot 10^{-1}$	2.33$\cdot 10^{-4}$	5.83$\cdot 10^{-4}$	6.03$\cdot 10^{-1}$	1.65$\cdot 10^{-4}$	5.27$\cdot 10^{-4}$
1.3	0.01	5.85$\cdot 10^{-1}$	1.63$\cdot 10^{-5}$	1.57$\cdot 10^{-4}$	9.68$\cdot 10^{-1}$	1.43$\cdot 10^{-5}$	5.46$\cdot 10^{-4}$
	0.05	5.41$\cdot 10^{-1}$	5.31$\cdot 10^{-5}$	4.45$\cdot 10^{-4}$	9.21$\cdot 10^{-1}$	7.35$\cdot 10^{-5}$	5.79$\cdot 10^{-4}$
	0.1	4.85$\cdot 10^{-1}$	2.63$\cdot 10^{-4}$	6.18$\cdot 10^{-4}$	8.71$\cdot 10^{-1}$	1.67$\cdot 10^{-4}$	6.84$\cdot 10^{-4}$

Three types of SNPs have been simulated: *(i)* trait differential SNPs; *(ii)* null SNPs; and *(iii)* stress SNPs. . Under each condition, we simulated individual level genotype with these three types of SNPs for *N* cases and *N* controls in each study ($N = 2000$ and $N=5000$) and generated GWAS summary statistics for each study. and generated GWAS summary statistics for each study respectively. We subsequently used the summary statistics to run cc-GWAS in ReACt. We reported the power for detecting type *(i)* SNPs, and false positive rates for picking up type *(ii)* SNPs (Type I err.$^{\texttt {(ii)}}$) and type *(iii)* SNPs (Type I err.$^{\texttt {(iii)}}$) under a significance threshold $p < 5 \cdot 10^{-5}$.

Next, we evaluated the performance of our method on real GWAS summary statistics and compared our method with the recently released method of^16^. We analyzed BIP^32^ and SCZ^33^ datasets, for which case-case GWAS with individual level data was available^34^. We filtered out SNPs that showed untrustworthy estimates of the stratification effect (${{\mathrm {SE}}}_s > 0.05$, see “cc-GWAS using summary statistics” section for details). This reduced our output size from 8,983,436 SNPs being analyzed to 7,110,776 SNPs. Out of those, our analysis revealed a total of 18 genome-wide significant risk loci, including the two regions identified by^34^, namely regions 1q25.1 and 20q13.12). We compared our statistics for SNPs that were also analyzed in^16^ and results for this comparison are shown in Table  4. The two cc-GWAS methods are mostly comparable. By definition, both we and Peyrot et al.^16^ only used summary statistics as input, and could not apply the individual level quality control steps of^34^. As a result, both methods identified additional significant loci showing divergent genetic effects between BD and SCZ compared to^34^, mainly due to a much larger effective sample size. Results for all genome-wide significant risk loci are shown in Table S6.

**Table 4:** Comparison of genomic regions showing significant divergent genetic effects between BD and SCZ as detected by ReACt and ccGWAS by Peyrot et al.^16^.

Region			Our method (ReACt)			ccGWAS		
CHR	Start	End	SNP	BP	*p*-value	SNP	BP	*p*-value ($P_{OLS}$)
1	50826176	51118253	rs6682989	50826176	$\varvec{3.08 \cdot 10^{-8}}$	–	–	6.10 $\cdot 10^{-7}$
1	98325796	98559093	rs2660304	98512127	$\varvec{4.20 \cdot 10^{-9}}$	–	–	$\varvec{2.20 \cdot 10^{-9}}$
**1**	**173867252**	**174643725**	rs6701877	174015259	$\varvec{4.02 \cdot 10^{-8}}$	–	–	$\varvec{5.80 \cdot 10^{-10}}$
2	27498734	27752296	rs113954968	27696207	$\varvec{2.93\cdot 10^{-8}}$	–	–	1.10$\cdot 10^{-6}$
3	62563175	62583180	rs1993149	62572944	$\varvec{2.10\cdot 10^{-8}}$	–	–	8.10$\cdot 10^{-7}$
3	135807609	136597120	rs9866687	94828190	6.55$\cdot 10^{-7}$	–	–	$\varvec{4.00\cdot 10^{-8}}$
3	135807609	136597120	rs7372313	135872958	$\varvec{1.02\cdot 10^{-8}}$	rs1278493	135814009	$\varvec{1.20\cdot 10^{-8}}$
7	28453906	28484317	rs2192303	28478332	$\varvec{3.57\cdot 10^{-8}}$	rs7790864	28478625	$\varvec{2.20\cdot 10^{-8}}$
8	27406353	27453579	rs11778040	27419807	5.39$\cdot 10^{-7}$	–	–	$\varvec{4.80\cdot 10^{-8}}$
9	23345347	23362311	rs12554512	23352293	$\varvec{3.58\cdot 10^{-10}}$	–	–	$\varvec{4.10\cdot 10^{-8}}$
9	36894685	36963222	rs2039142	36963222	$\varvec{1.95\cdot 10^{-8}}$	–	–	2.10$\cdot 10^{-6}$
10	353306	418676	rs35198327	354301	$\varvec{7.69\cdot 10^{-9}}$	–	–	1.10$\cdot 10^{-7}$
12	108596308	108633649	rs3764002	108618630	$\varvec{3.28\cdot 10^{-9}}$	–	–	$\varvec{6.30\cdot 10^{-11}}$
12	110294902	111212762	rs28637922	110819139	$\varvec{5.11\cdot 10^{-10}}$	–	–	$\varvec{8.10\cdot 10^{-12}}$
16	79386766	79463881	rs6564668	79457393	$\varvec{1.86\cdot 10^{-8}}$	rs9319540	79458022	$\varvec{3.70\cdot 10^{-8}}$
19	1812521	1866427	rs1054972	1852582	6.43$\cdot 10^{-8}$	–	–	$\varvec{1.80\cdot 10^{-8}}$
**20**	**47511792**	**47938833**	rs6095394	47625544	$\varvec{1.43\cdot 10^{-9}}$	rs11696888	47753265	$\varvec{1.40\cdot 10^{-9}}$

We carried out cc-GWAS with ReACt using summary statistics of BD and SCZ and compared our results with the results from Peyrot et al. Only SNPs that are analyzed in both studies are included for the comparison. Genomic regions that are identified to show significant divergent genetic effects between BD and SCZ in either result are shown. CHR, Start and End are chromosomal and base-pair ranges for the region; SNP, BP and *p*-value (ordinary least squares *p*-values, $P_{OLS}$, for ccGWAS by Peyrot et al.) are properties of the leading SNP (if the regions is reported genome-wide significant) or statistics for the matching SNP (if the region is not reported as genome-wide significant, but is detected by the other method); *p*-values in bold are leading SNPs that are reported genome-wide significant by each method; Regions with CHR, Start and End in bold are two loci that were also identified by the case-case GWAS using individual level data^34^.

### Group PRS

#### Our approach

We realized that our new method opens up a new opportunity for summary-statistics-based analysis which was not possible before: even though we still cannot compute individual level PRS without access to raw genotypes, we observe that, under the additive model, the mean and standard deviation of PRS for a population are just functions of SNP allele frequencies in the target group (see “cc-GWAS and group PRS” section for details). Therefore, the novel summary-statistics-based framework for analysis, which returns estimates of allele frequencies for cases and controls using GWAS summary statistics, also allows us to estimate means and standard deviations of PRS for case and control groups using the GWAS summary statistics of the target study. With such information (and a fair assumption of normality in the underlying PRS distribution), we can further run a *t*-test in order to get a *p*-value comparing the difference of PRS between cases and controls.

More specifically, in the additive model, the mean and variance of PRS for a population can be expressed as follows:

$$\begin{aligned}&{{\mathrm {mean}}}({\text {PRS}}) = \frac{\sum _{i=1}^M S_i p_{i}}{M},\text { and }\\&{{\mathrm {Var}}}(\text {PRS}) = \frac{\sum _{i=1}^M S_i^2 p_iq_i}{2M^2}. \end{aligned}$$

In the above $S_i$ is the weight of SNP *i* inferred from the base summary statistics (typically $S_i = \frac{\log (OR_i)}{SE_i}$), *M* is the total number of SNPs used in the PRS computation, and $p_{i}$ and $q_{i} = 1-p_{i}$ are allele frequencies of the effective allele and the non-effective allele for SNP *i*. Therefore, we can simply use the allele frequencies of cases and controls that were computed in “Mathematical foundations” section in order to get the mean and variance of PRS in cases and controls. See “cc-GWAS and group PRS” section for details.

#### Group PRS: performance evaluation

We first tested our methods on synthetic data without any confounding factors (ie., no stratification). After generating GWAS summary statistics for synthetic base and target datasets, we compared the estimated group means and standard deviations using our method (which operates on summary statistics) with the real group means and standard deviations of PRS computed from the individual level genotypes using PRSice2^35^. The results successfully proved that in this scenario our method is extremely accurate. See Table 5 which shows typical representative results from our experimental evaluations; essentially identical results were observed in all our experiments on synthetic data.

**Table 5:** Estimated and real group mean and standard deviation of PRS for a synthetic target population.

Risk	Group	Our Method (ReACt)		PRSice2	
		Est. group mean	Est. group sd	Real group mean	Real group sd
1.15	Cases	0.0009	0.0078	0.0009	0.0076
	Controls	− 0.0037	0.0078	− 0.0036	0.0081
1.2	Cases	0.0016	0.0060	0.0016	0.0059
	Controls	− 0.0065	0.0060	− 0.0064	0.0061
1.3	Cases	0.0021	0.0041	0.0021	0.0040
	Controls	− 0.0125	0.0041	− 0.0125	0.0040

We compared group mean and standard deviation of PRS estimated by ReACt from summary statistics of synthetic base and target studies to the real group mean and standard deviation of individual level PRS obtained using summary statistics of the base and individual level genotype of the target computed by PRSice2. Est stands for estimated. Note that the synthetic data is not subject to clumping since the simulation model does not generate LD structure.

We further tested our method on real GWAS data, using GWAS summary statistics for MDD^36^ as the base study and assessing its predicting power on 18,368 *independent* depressive episode cases and 312,849 ancestry-matched controls in UK biobank. We did not choose the latest MDD GWAS to be a base study because the latest one has included samples from UK biobank. To run ReACt, we generated GWAS summary statistics for the target dataset as described. We compared the estimated PRS statistics using our methods with the real PRS statistics computed using PRSice2. The results are shown in Table 6; note that since real GWAS datasets are subject to within study population stratification, we did not expect our method to be as accurate as it was on synthetic data without such stratification. There was, however, very high concordance between the results returned by our methods and ground truth. Finally, we applied our methods on summary statistics of eight psychiatric disorders. We evaluated their pairwise PRS predictive power by estimating *t*-test *p*-values. For this experiment, we took into account potential sample overlap between all pairs of base and target studies; see Section 5.3 in supplementary text for details of our sample overlap correction procedure. Results are shown in Table 7 and we observe that, in general, our results coincide with pairwise genetic correlation between disorders as discussed in^7^.

**Table 6:** Estimated and real group mean and standard deviation of PRS for depressive episode cases and controls in UK biobank population.

*P*-thres	#SNPs	Trait	Our method (ReACt)		PRSice2					
			*t*-test		*t*-test		Reg. w/o covatiate		Reg. w/top 5PCs	
			Mean PRS	*p*-val	Mean PRS	*p*-val	$r^2$	*p*-val	$r^2$	*p*-val
0.1	4236	Cases	$-$0.0023	5.50$\cdot 10^{-3}$	$-$0.0023	$3.97 \cdot 10^{-3}$	$2.48\cdot 10^{-5}$	$4.18 \cdot 10^{-3}$	$3.54 \cdot 10^{-5}$	$4.14 \cdot 10^{-3}$
		Controls	$-$0.0023		$-$0.0024					
0.01	594	Cases	$-$0.0036	1.47$\cdot 10^{-3}$	$-$0.0032	$1.42 \cdot 10^{-3}$	$3.06 \cdot 10^{-5}$	1.45 $\cdot 10^{-3}$	$4.35 \cdot 10^{-5}$	1.44 $\cdot 10^{-3}$
		Controls	$-$0.0036		$-$0.0032					
0.001	82	Cases	0.0112	1.09$\cdot 10^{-1}$	0.0147	$1.54 \cdot 10^{-1}$	$6.17 \cdot 10^{-6}$	1.53 $\cdot 10^{-1}$	$3.19 \cdot 10^{-5}$	$1.51 \cdot 10^{-1}$
		Controls	0.0112		0.0146					
$10^{-4}$	10	Cases	$-$0.0244	9.36$\cdot 10^{-2}$	$-$0.0247	$1.16 \cdot 10^{-1}$	$7.57 \cdot 10^{-6}$	1.13 $\cdot 10^{-1}$	$2.96 \cdot 10^{-5}$	$1.12 \cdot 10^{-1}$
		Controls	$-$0.0246		$-$0.0249					

We assessed the performance of our method using the summary statistics of an independent MDD GWAS as the base study, and the UK biobank samples, including 18,368 cases with depressive episode and 312,849 controls, as the target population. We generated summary statistics for the target populations and estimated group mean PRS and standard deviation of target PRS using ReACt. We computed the individual level PRS for the target study using PRSice2. For both methods, we computed PRS using independent SNPs from the base summary statistics with *p*-values below various thresholds (*P*-thres) and compared the performances under each threshold. For ReACt, mean PRS represents the estimated group mean PRS for cases and controls; *p*-val are the *t*-test *p*-values comparing PRS distribution in cases and in controls. For PRSice2, mean PRS represents real group mean PRS computed from individual level data and *p*-val are the *t*-test *p*-values comparing real PRS distribution in cases and in controls; reg. w/o covariate indicates regression results without covariates, which include the regression $r^2$ value (reg. $r^2$) and the *p*-value for the PRS predictor (*p*-val); reg. w/top 5PCs indicates the regression results including the top five PCs as covariates, which also included the regression $r^2$ value (reg. $r^2$) and the *p*-value for the PRS predictor (*p*-val).

**Table 7:**
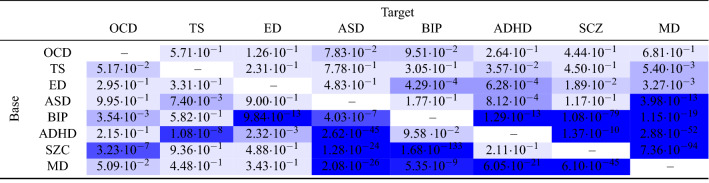
Using our method to perform PRS comparisons across eight neuropsychiatric disorders.

We further applied our method to the summary statistics of eight neuropsychiatric disorders from PGC (see table 6 for details). For each disorder, we used PGC GWAS summary statistics to compute the group mean and standard deviation of PRS for the other seven disorders. All group PRS were estimated using independent SNPs with $p < 10^{-5}$in the base summary statistics. We report *p*-values from a *t*-test comparing the group mean PRS of cases against controls in the target study, and cells with deeper blue colors correspond to lower *p*-values. The threshold of significance under multiple testing correction is $p < 8.93 \cdot 10^{-4}$.

## Discussion

Extracting as much information as possible from easily accessible GWAS summary statistics can help accelerate research that aims to elucidate the genetic background of complex disease, allowing fast sharing of results and datasets while alleviating privacy concerns. In prior work, GWAS meta-analyses and cc-GWAS were treated as separate tasks with different theoretical foundations. In our work, we compare and evaluate leading methods and present a novel framework that unifies analyses under the same methodological umbrella, while expanding capabilities of summary-statistics-based analysis even further allowing, for the first time, group PRS estimation. Our methods do not affect the differential privacy established by sharing GWAS summary statistics^37^. Moreover, as our allelic frequency reconstruction framework does not make any assumptions, our approach is unlikely to introduce additional bias into the results. However, just like any other summary-statistics-based method, it is still possible that the performance of ReACt might get affected by preexisting ascertainment bias from the input GWAS.

In terms of GWAS meta-analysis, we found that all three methods we tested are comparable in terms of power and type I error rates. However, both METAL and ReACt greatly outperform ASSET in terms of running time. The reconstruction of the allelic counts for each SNP in ReACt allows us to run a full logistic regression model instead of doing the conventional inverse-variance weighted fixed-effect meta-analysis, under the assumption of HWE. Our results on real GWAS data showed that just the standard HWE filtering threshold of $10^{-6}$ is needed, which is a typical quality control step in any GWAS. Note that this assumption is only used when we calculate genotype frequencies from the allelic frequencies, which is the case only in the fixed effect meta-analysis part of our work. Future work could explore whether we can further relax this threshold, or even remove this assumption. Our approach shows increased power in experiments on synthetic data, especially in cases where there is larger $F_{st}$ difference between the input studies, and provides robust results in real GWAS settings. One of the biggest concerns in GWAS meta-analysis is sample overlap between different studies. ASSET only allows correction for *known* sample overlap, whereas METAL’s development version is able to correct for *unknown* sample overlap. Our work here presents, for the first time, a thorough evaluation of correction for known and unknown sample overlap; our sample overlap correction is theoretically founded and more intuitive compared to previous methods^27^.

We further propose a novel perspective on case-case association studies (cc-GWAS), allowing analysis without the need for complicated assumptions or side information apart from sample sizes. To the best of our knowledge, the only prior work on summary statistics based case-case GWAS is^16^. In our work, we achieve this objective in a straightforward manner: we directly compare the reconstructed allele frequencies of each SNP in two groups of cases, without the need to estimate heritabilities or prevalence of disorders as in^16^. The fact that case-case GWAS using ReACt analyzes each SNP independently also allows the user to run the analysis even when only a subset of the GWAS results are made available, which is a common scenario in practice and could not be addressed by prior work. Further, we do not need any extra assumptions on the distribution of SNP effects.

ReACt showed good control of type I errors in null SNPs (type II SNPs) given sufficiently large control sample sizes for both input studies. In practice, our experiments demonstrated that we can get accurate results with 2000 controls from each input GWAS, which is a reasonable sample size in modern GWAS. It also shows slightly higher, but under-controlled, type I errors in the stress test SNPs (type III SNPs), which is also observed by the method of^16^. As also pointed out by^16^, we do not expect the existence of stress SNPs to be particularly common in practice.

A notable difference between our method and the work of^16^ is that we do not filter for SNPs showing association due to differential tagging effects. While analyzing such SNPs, our method behaves more like a direct case-case GWAS using individual level data.

Our framework also introduces a novel perspective on case-control PRS. Conventionally, PRS for a target study is only accessible from individual level genotype data. However, we notice that the group means and standard errors of PRS can in fact be estimated using only summary statistics of both the base and target studies. With such statistics available, a *t*-test can be carried out instead place of logistic regression, which is commonly used for predictability evaluation when the individual level PRS are available. It is worth noting that, for case-control studies, *t*-tests and logistic regression are testing the same hypothesis: whether scores generated from the SNP effect of a base study can differentiate individuals in the target study, or, equivalently, whether the base study can predict the case/control status of samples in the target study. We applied our method to summary statistics of eight psychiatric disorders from PGC for predicting group PRS and found the results in general concordance with the genetic correlation obtained by the work of Lee et al.^7^.

In our work, PRS evaluations use the *p*-value based clumping and thresholding (PC+T) approach. However, the methodology underlying the ReACt group PRS can be easily adapted to any other PRS computation model, e.g.,^38^ (SBLUP^39^, LDpred^40^ , PRS-CS^41^, SBayesR^42^ and other Bayesian based methods^43^ etc).

It is worth noting that given SNP effect sizes and weights as input, ReACt does not require the base summary statistics to be from a case-control GWAS because, in PRS computations, the base summary statistics provide the predictor weights and we do not need to convert them back into allele frequencies. This fact makes ReACt easily applicable on any of the aforementioned SNP re-weighting schemes. To date, most PRS improvements target the selection and prioritization of SNPs or the adjusting of the weights to build a better prediction model using the base study. Our work contributes from a different perspective: it allows the user to evaluate the performance of models without access to individual level genotype data. Moreover, results from group PRS using our approach can be further connected with^22^ to quantify the predisposition to a particular disorder that is explained by a certain SNP set. Finally, a notable feature of ReACt is that, theoretically, it can handle known and unknown sample overlap between base and target populations; to the best of our knowledge, this is done for the first time for PRS computations. Sample overlap has long been known as a problem in PRS and our approach provides a good starting point for future work. We do note that, recently, a different method has also been proposed to specifically correct the inflation due to known sample overlap between base and target studies in PRS evaluations with individual level data^44^. An interesting future research direction would be to combine the results of^44^ with summary statistic methods such as ReACt.

Our framework is robust against within-study stratification effects. However, users should keep in mind that general rules of thumb for conventional PRS also apply to our method. For instance, the SNPs used for PRS computations are expected to be independent to a certain extent (clump/prune/LASSO shrink the summary statistics)^19^ and the predictive power of output PRS will be subject to the power of the base study^21^ and the *p*-value threshold chosen by the user.

Our work opens many future research directions. First, the reconstruction scheme that our framework is built upon is based on input summary statistics that are generated using a logistic regression or a $\chi ^2$-test. We have not yet explored how to adapt our framework to operate on summary statistics from other models. Theoretically, all we need is GWAS summary statistics that can be converted into *OR* and *SE* for each SNP. There exist summary statistics-based methods transforming GWAS results obtained from linear mixed model association to odds ratio^45^, and it will be interesting to further explore how such methods could interface with our approach. Also, our meta-analysis module only investigated the most straight forward application of ReACt in a fixed-effect model. It would be interesting to explore methodologies that carry out random-effect meta-analyses using the same framework. Another interesting topic for future work would be to incorporate information beyond GWAS summary statistics. For example, one could consider incorporating external information such as LD structure using LD reference maps, or eQTL and SNP to gene annotations. Such information could be used to improve the accuracy of sample overlap estimation and to extend the group-PRS applications. Furthermore, although outside the scope of our analysis here, one could investigate expanding towards methods that perform haplotype (instead of genotype) reconstruction. Overall, our work here highlights the power of summary-statistics-based methodology and opens up additional avenues for research.

## Methods

### Our framework

#### Notation

Prior to introducing our methods, we discuss notational conventions. We will reserve the subscript *i* to denote SNP number: given, say, *M* SNPs, *i* will range between one and *M*. Similarly, we will reserve the subscript $\ell$ to denote the study number: given *L* studies from which summary statistics will be meta-analyzed, $\ell$ will range between one and *L*. We assume that all *L* studies released summary statistics on a *common set* of *M* SNPs. For simplicity, we will first describe our methods for the case $L=2$ (i.e., when exactly two studies are jointly meta-analyzed) and we will generalize our approach in “Meta-analyzing multiple datasets” section for $L > 2$.

We will use the three-letter shorthand cse for cases and the three-letter shorthand cnt for controls. We reserve the variable *a* to represent counts of the effective allele and the variable *u* to represent counts of the non-effective allele. We also reserve the variable *N* to represent counts for the number of cases or controls. Given the above conventions, we now present the following table of allele counts (effective and non-effective allele) for SNP *i* ($i=1\ldots M$) in study $\ell$ ($\ell =1\ldots L$) (Table 8).

**Table 8:** Table of allele counts for SNP *i* ($i=1\ldots M$) in the $\ell$ -th GWAS ($\ell =1\ldots L$).

	$A_1$ (effective allele)	$A_2$ (non-effective allele)	Number of alleles
Cases	$a_{i\ell }^{\text {cse}}$	$u_{i\ell }^{\text {cse}}$	$2N^{\text {cse}}_{\ell }$
Controls	$a_{i\ell }^{\text {cnt}}$	$u_{i\ell }^{\text {cnt}}$	$2N^{\text {cnt}}_{\ell }$

The total number of cases for the $\ell$-th study is $N_{\ell }^{{\texttt {cse}}}$ and the total number of controls for the $\ell$-th study is $N_{\ell }^{{\texttt {cnt}}}$. Clearly, the total number of cases and controls in a study is the same for all SNPs, which is why the variable *N* does not depend on *i*. The total number of alleles in cases and controls is equal to twice the number of cases and controls, respectively.

Using the above table, we can also compute the frequencies of the effective or non-effective allele in cases and controls. Table 9 summarizes frequency notation for SNP *i* ($i=1\ldots M$) in study $\ell$ ($\ell =1\ldots L$).

**Table 9:** Notations and definitions of (effective or non-effective) allele frequencies in cases and controls.

$p_{i \ell }^{{\texttt {cse}}} = \frac{a_{i\ell }^{\text {cse}}}{a_{i\ell }^{\text {cse}}+u_{i\ell }^{\text {cse}}}$	Frequency of the *effective allele* $A_1$ in cases
$p_{i \ell }^{{\texttt {cnt}}} = \frac{a_{i\ell }^{\text {cnt}}}{a_{i\ell }^{\text {cnt}}+u_{i\ell }^{\text {cnt}}}$	Frequency of the *effective allele* $A_1$ in controls
$q_{i \ell }^{{\texttt {cse}}} = \frac{u_{i\ell }^{\text {cse}}}{a_{i\ell }^{\text {cse}}+u_{i\ell }^{\text {cse}}}$	Frequency of the *non-effective allele* $A_2$ in cases
$q_{i \ell }^{{\texttt {cnt}}} = \frac{u_{i\ell }^{\text {cnt}}}{a_{i\ell }^{\text {cnt}}+u_{i\ell }^{\text {cnt}}}$	Frequency of the *non-effective allele* $A_2$ in controls

The subscripts *i* and $\ell$ indicate SNP number and study number, respectively.

Obviously,

$$\begin{aligned} p_{i \ell }^{{\texttt {cse}}}+q_{i \ell }^{{\texttt {cse}}}&=1\\ p_{i \ell }^{{\texttt {cnt}}}+q_{i \ell }^{{\texttt {cnt}}}&=1. \end{aligned}$$

#### Reconstructing allele counts

Using Table 8, notice that the odds ratio (OR) and its corresponding standard error (SE) for SNP *i* in study $\ell$ are given by the following formulas:

1 $$\begin{aligned} OR_{i\ell }&= \frac{a_{i\ell }^{\text {cse}} \cdot u_{i\ell }^{\text {cnt}}}{a_{i\ell }^{\text {cnt}} \cdot u_{i\ell }^{\text {cse}}}, \end{aligned}$$

2 $$\begin{aligned} SE_{i \ell }&= \sqrt{\frac{1}{a_{i\ell }^{\text {cse}}} + \frac{1}{u_{i\ell }^{\text {cse}}} + \frac{1}{a_{i\ell }^{\text {cnt}}} + \frac{1}{u_{i\ell }^{\text {cnt}}}}. \end{aligned}$$

Additionally,

3 $$\begin{aligned} 2N^{\text {cse}}_{\ell }&= a_{i\ell }^{\text {cse}} + u_{i\ell }^{\text {cse}},\quad \text {and} \end{aligned}$$

4 $$\begin{aligned} 2N^{\text {cnt}}_{\ell }&= a_{i\ell }^{\text {cnt}} + u_{i\ell }^{\text {cnt}}. \end{aligned}$$

By solving the system of non-linear Eqs. (1), (2), (3), and (4), we can recover $a_{i\ell }^{\text {cse}}$, $u_{i\ell }^{\text {cse}}$, $a_{i\ell }^{\text {cnt}}$, and $u_{i\ell }^{\text {cnt}}$ for SNP *i* in study $\ell$. Notice that $OR_{i\ell }$, $SE_{i\ell }$, $N^{\text {cse}}_{\ell }$, and $N^{\text {cnt}}_{\ell }$ are available from summary statistics. See Appendix 5.2 for details on solving the aforementioned system of non-linear equations.

#### Reconstructing genotype counts

Given the reconstructed allele counts of “Reconstructing allele counts” section, we can now reconstruct genotype counts for SNP *i* in the $\ell$-th study. In order to do this, we need to assume that SNP *i* is in HWE in both case and control groups of study $\ell$. Note that a well-performed GWAS should have SNPs drastically violating HWE filtered out. As demonstrated in our results, SNPs with HWE *p*-value larger than $10^{-6}$ (a common threshold applied in most GWAS) do not affect the performance of ReACt in practice. More precisely, assume that for SNP *i* in study $\ell$ we have reconstructed its allele table count (Table 8). Then, by assuming that this SNP is in HWE in study $\ell$, we can compute the number of cases and controls that exhibit a particular genotype. Recall that there are three possible genotypes: $A_1A_1$, $A_1A_2$, and $A_2A_2$. We will represent each genotype by counting the number of copies of the effective allele in each genotype. Thus, $A_1A_1$ will correspond to two, $A_1A_2$ will correspond to one, and $A_2A_2$ will correspond to zero.

Following our notational conventions from “Notation” section, we can now compute the entries in Table 10 of genotype counts for SNP *i* in study $\ell$.

**Table 10:** Genotype counts for cases and controls for SNP *i* in study $\ell$.

	$A_1A_1$ (two copies of $A_1$)	$A_1A_2$ (one copy of $A_1$)	$A_2A_2$ (zero copies of $A_1$)
Cases	$N_{i\ell }^{\text {cse}}(2)=(p_{i \ell }^{{\texttt {cse}}})^2 N^{{\texttt {cse}}}_{\ell }$	$N_{i\ell }^{\text {cse}}(1) = 2p_{i \ell }^{{\texttt {cse}}} q_{i \ell }^{{\texttt {cse}}} N^{{\texttt {cse}}}_{\ell }$	$N_{i\ell }^{\text {cse}}(0) = (q_{i \ell }^{{\texttt {cse}}})^2 N^{{\texttt {cse}}}_{\ell }$
Controls	$N_{i\ell }^{\text {cnt}}(2)=(p_{i \ell }^{{\texttt {cnt}}})^2 N^{{\texttt {cnt}}}_{\ell }$	$N_{i\ell }^{\text {cnt}}(1) = 2p_{i \ell }^{{\texttt {cnt}}} q_{i \ell }^{{\texttt {cnt}}} N^{{\texttt {cnt}}}_{\ell }$	$N_{i\ell }^{\text {cnt}}(0) = (q_{i \ell }^{{\texttt {cnt}}})^2 N^{{\texttt {cnt}}}_{\ell }$

Using the above formulas, we can reconstruct the genotype counts for cases and controls for each of the three possible genotypes.

It is worth noting that

5 $$\begin{aligned} N_{\ell }^{\text {cse}}&= N_{i\ell }^{\text {cse}}(0) + N_{i\ell }^{\text {cse}}(1) + N_{i\ell }^{\text {cse}}(2), \end{aligned}$$

6 $$\begin{aligned} N_{\ell }^{\text {cnt}}&= N_{i\ell }^{\text {cnt}}(0) + N_{i\ell }^{\text {cnt}}(1) + N_{i\ell }^{\text {cnt}}(2). \end{aligned}$$

Next, we reconstruct the genotype vector for SNP *i* in study $\ell$ as follows:

$$\begin{aligned} {\mathbf {g}}_{i\ell } = \left[ \begin{array}{cccccc} \underbrace{0\ldots 0}_{N_{i\ell }^{\text {cse}}(0)}&\underbrace{1\ldots 1}_{N_{i\ell }^{\text {cse}}(1)}&\underbrace{2\ldots 2}_{N_{i\ell }^{\text {cse}}(2)}&\underbrace{0\ldots 0}_{N_{i\ell }^{\text {cnt}}(0)}&\underbrace{1\ldots 1}_{N_{i\ell }^{\text {cnt}}(1)}&\underbrace{2\ldots 2}_{N_{i\ell }^{\text {cnt}}(2)} \end{array}\right] . \end{aligned}$$

Using Eqs. (5) and  (6), it is easy to conclude that the vector ${\mathbf {g}}_{i\ell }$ has a total of

$$\begin{aligned} N_{\ell }^{\text {cse}}+N_{\ell }^{\text {cnt}} \end{aligned}$$

entries, which is equal to the number of samples (cases plus controls) included in the $\ell$-th study. We can also form the response vector ${\mathbf {y}}_{\ell }$ for the $\ell$-th study, indicating whether a sample is a case (i.e., one) or a control (i.e., zero) as follows:

7 $$\begin{aligned} {\mathbf {y}}_{\ell } = \left[ \begin{array}{cc} \underbrace{1\ldots 1}_{N_{\ell }^{\text {cse}}}&\underbrace{0\ldots 0}_{N_{\ell }^{\text {cnt}}} \end{array}\right] . \end{aligned}$$

Note that the vectors ${\mathbf {y}}_{\ell }$ and ${\mathbf {g}}_{i\ell }$ have the same dimensions (same number of entries). It should be clear that the vector ${\mathbf {y}}_{\ell }$ *is the same for all SNPs* in the $\ell$-th study and hence does not depend on the SNP number *i*.

We conclude the section by discussing the construction of an indicator vector ${\mathbf {s}}$ that will denote the study from which a particular sample in our meta-analysis originated. For the sake of simplicity, assume that we meta-analyze summary statistics from two studies ($L=2$). Then, following the above discussion, we can construct the genotype vectors ${\mathbf {g}}_{i1}$ and ${\mathbf {g}}_{i2}$ and concatenate them to construct the overall genotype vector for the *i*-th SNP in both studies:

$$\begin{aligned} {\mathbf {g}}_i = \left[ {\mathbf {g}}_{i1} \ {\mathbf {g}}_{i2}\right] . \end{aligned}$$

Similarly, we can construct the overall response vector ${\mathbf {y}}$ for both studies:

$$\begin{aligned} {\mathbf {y}} = \left[ {\mathbf {y}}_{1} \ {\mathbf {y}}_{2}\right] . \end{aligned}$$

Notice that the vectors ${\mathbf {g}}_i$ and ${\mathbf {y}}$ have the same dimensions (number of entries), equal to the number of samples (cases plus controls) in both studies, i.e., equal to

$$\begin{aligned} N = N_{1}^{\text {cse}}+N_{1}^{\text {cnt}}+N_{2}^{\text {cse}}+N_{2}^{\text {cnt}}. \end{aligned}$$

We can now construct the indicator vector ${\mathbf {s}}$ as follows:

$$\begin{aligned} {\mathbf {s}} = \left[ \begin{array}{cc} \underbrace{0\ldots 0}_{N_{1}^{\text {cse}}+N_{1}^{\text {cnt}}}&\underbrace{1\ldots 1}_{N_{2}^{\text {cse}}+N_{2}^{\text {cnt}}} \end{array}\right] . \end{aligned}$$

Note that a value of zero in ${\mathbf {s}}$ indicates that the corresponding sample belongs to the first study while a value of one in ${\mathbf {s}}$ indicates that the corresponding sample belongs to the second study.

### Fixed-effect meta-analysis

#### Logistic regression

We run logistic regression for each SNP separately; recall that we number SNPs in our meta-analysis from one up to *M*. For notational convenience and since we run logistic regression in an identical manner for each SNP, without loss of generality we focus on a single SNP. Let the genotype vector for the selected SNP be denoted by ${\mathbf {g}}$; let ${\mathbf {s}}$ be the study indicator vector; and let ${\mathbf {y}}$ be the response vector, as discussed in the previous section. Recall that all three vectors have the same dimensions (same number of entries), equal to *N*, namely the total number of cases and controls in both studies. *Notice that we dropped the subscript* *i* *from the vector* ${\mathbf {g}}$ *for notational convenience, since our discussion in this section will focus on a fixed SNP* *i*, *without loss of generality.*

Using notation from the previous section, while dropping the subscript *i* from the genotype vector ${\mathbf {g}}$, allows us to formulate logistic regression as follows:

8 $$\begin{aligned} {{\mathsf {Pr}}}({\mathbf {y}}_j = 1 | {\mathbf {g}}_j,{\mathbf {s}}_j) = S(\beta _0 + \beta _1 {\mathbf {g}}_j + \beta _2 {\mathbf {s}}_j), \end{aligned}$$

where $S(x) = (1+e^{-x})^{-1}$ is the sigmoid function; ${\mathbf {y}}_j$ denotes the *j*th entry of the vector ${\mathbf {y}}$; ${\mathbf {s}}_j$ denotes the *j*th entry of the vector ${\mathbf {s}}$; and $\beta _0$, $\beta _1$, and $\beta _2$ are the unknown coefficients of the logistic regression formulation. Here $\beta _0$ corresponds to the constant offset, $\beta _1$ corresponds to the genotype, and $\beta _2$ corresponds to the study-of-origin. We also highlight that ${\mathbf {g}}_j$ denotes the *j*th entry of the vector ${\mathbf {g}}$; recall once again that we dropped the subscript *i* from the genotype vector in this section. The range for all subscripts *j* for the above vectors is between one and *N*.

In order to further describe how logistic regression was implemented in our experiments, it will be convenient to introduce additional notation. Let $\mathbf {\beta }$ be the vector

$$\begin{aligned} \mathbf {\beta }^T&= [\beta _0\ \beta _1\ \beta _2], \end{aligned}$$

and let ${\mathbf {x}}$ be the vector

$$\begin{aligned} {\mathbf {x}}_j^T&= [1\ {\mathbf {g}}_j\ {\mathbf {s}}_j]. \end{aligned}$$

Thus, $\mathbf {\beta }$ is the vector of the (unknown) logistic regression coefficients, while ${\mathbf {x}}_j^T$ for all $j=1\ldots N$ is the vector representing the constant offset, the genotype, and the study origin for the *j*th sample in our meta-analysis. This allows us to rewrite Eq. (8) as follows:

$$\begin{aligned} {{\mathsf {Pr}}}({\mathbf {y}}_j = 1 | {\mathbf {g}}_j,{\mathbf {s}}_j) = S(\mathbf {\beta }^T \cdot {\mathbf {x}}_j). \end{aligned}$$

We can now compute the negative log-likelihood (NLL) function for $\mathbf {\beta }$ as follows:

$$\begin{aligned} NLL(\mathbf {\beta })&= -\sum _{j=1}^N \log ({{\mathsf {Pr}}}({\mathbf {y}}_j)) = 1 | {\mathbf {x}}_j) \\&= -\sum _{j=1}^{N}{\mathbf {y}}_j \log S(\mathbf {\beta }^T \cdot {\mathbf {x}}_j)+(1-{\mathbf {y}}_j) \log (1-S(\mathbf {\beta }^T \cdot {\mathbf {x}}_j)). \end{aligned}$$

Thus, $\mathbf {\beta }$ can be estimated using the Iterative Re-weighted Least Squares (IRLS) algorithm^46^ as follows:

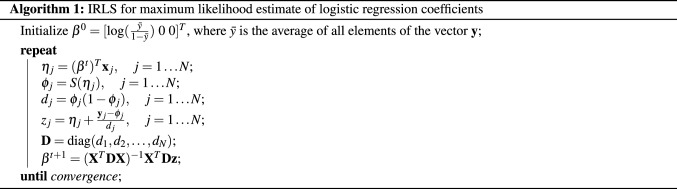



In the IRLS algorithm, we let ${\mathbf {D}}$ denote the diagonal $N \times N$ matrix whose diagonal entries are $d_1,d_2,\ldots ,d_N$; we let ${\mathbf {X}}$ denote the $N \times 3$ matrix whose rows are the vectors ${\mathbf {x}}_j^T$ for $j=1\ldots N$; and we let ${\mathbf {z}}$ denote the vector whose entries are the $z_j$ for $j=1\ldots N$. Using this notation, the matrix ${\mathbf {H}} = {\mathbf {X}}^T{\mathbf {D}}{\mathbf {X}}$ is the $3 \times 3$ Hessian matrix of this logistic regression problem. The algorithm iterates over $t=0,1,2,\ldots$ and terminates when our convergence criterion, namely the difference $\Vert \mathbf {\beta }^{t+1}-\mathbf {\beta }^t\Vert$ (which is simply the sum of the absolute values of the three entries of the vector $\mathbf {\beta }^{t+1}-\mathbf {\beta }^t$) drops below the threshold $10^{-4}$, which is the same threshold as the one used by PLink^47^ for logistic regression.

Note that a drawback for logistic regression is that it can produce anti-conservative results under imbalance, which in our case, includes unbalanced sample sizes in cases and controls, as well as unbalanced sample sizes among input studies. We apply Firth bias-corrected logistic regression test^48,49^ to correct for the estimate under input imbalance (triggered when either the total case/control ratio, or maximum/minimum input sample size ratio is greater or equal to 5 by default). This approach has been reported with stable performance in both balanced and unbalanced studies, as well as with rare SNPs^50^.

We conclude this section by discussing how to compute a *p*-value for the logistic regression formulation of Eq. (8). First, it is well-known that the standard error for the three coefficients of the logistic regression formulation can be computed by using the inverse of the Hessian matrix ${\mathbf {H}}$. In particular, the standard error for $\mathbf {\beta }_0$ is equal to $SE_0 = \sqrt{(\mathbf {H^{-1}})_{11}}$; the standard error for $\mathbf {\beta }_1$ is equal to $SE_1 = \sqrt{(\mathbf {H^{-1}})_{22}}$; and the standard error for $\mathbf {\beta }_2$ is equal to $SE_2 = \sqrt{(\mathbf {H^{-1}})_{22}}$. As is typical in association studies, we focus on $SE_1$, the standard error for the vector of genotypes, and compute the respective *p*-value for the SNP-under-study using the Wald test. More specifically, we find the corresponding *p*-value of a *Z*-distribution for the parameter $\left| \frac{\beta _1}{SE_1}\right|$.

#### Correcting for sample overlap (two studies)

Sample overlap between studies can lead to an under-estimation of test statistics variance and results in an inflated test *p*-value. To prevent this from happening, we will use an “effective sample size” correction as follows. Assume that we are given Table 11, which details the number of overlapping samples between the two studies.

**Table 11:** Number of overlapping cases and controls between the two studies.

Overlapping	Study 2: case	Study 2: control
Study 1: case	$N_{{\texttt {shr}}}^{{\texttt {cse-cse}}}$	$N_{{\texttt {shr}}}^{{\texttt {cnt-cse}}}$
Study 1: control	$N_{{\texttt {shr}}}^{{\texttt {cse-cnt}}}$	$N_{{\texttt {shr}}}^{{\texttt {cnt-cnt}}}$

For example, the first cell of the table indicates the number of shared cases between the two studies. In practice, the off-diagonal cells of this table are close to zero, since they indicate cases in one study that became controls in the other study and vice-versa. Large numbers in these off-diagonal cells would indicate high heterogeneity across the two studies, in which case a fixed effect meta-analysis is not recommended.

Using the counts in Table 11, the number of shared cases between the two studies is equal to:

9 $$\begin{aligned} N_{{\texttt {shr}}}^{{\texttt {cse}}} = N_{{\texttt {shr}}}^{{\texttt {cse-cse}}} + \frac{N_{{\texttt {shr}}}^{{\texttt {cse-cnt}}}+N_{{\texttt {shr}}}^{{\texttt {cnt-cse}}}}{2}. \end{aligned}$$

Notice that if the off-diagonal entries in Table 11 are equal to zero then the above number reduces, obviously, to $N_{{\texttt {shr}}}^{{\texttt {cse-cse}}}$. Similarly, we have the number of shared controls equal to:

10 $$\begin{aligned} N_{{\texttt {shr}}}^{{\texttt {cnt}}} = N_{{\texttt {shr}}}^{{\texttt {cnt-cnt}}} + \frac{N_{{\texttt {shr}}}^{{\texttt {cnt-cse}}}+N_{{\texttt {shr}}}^{{\texttt {cse-cnt}}}}{2}. \end{aligned}$$

Then, the correction is simply carried out by multiplying the case/control sample size of each input study by a “deflation factor” defined as follows:

$$\begin{aligned} \lambda _\ell ^{{\texttt {cse}}}&= \frac{N_\ell ^{{\texttt {cse}}}}{N_\ell ^{{\texttt {cse}}}+N_{{\texttt {shr}}}^{{\texttt {cse}}}}\\ \lambda _\ell ^{{\texttt {cnt}}}&= \frac{N_\ell ^{{\texttt {cnt}}}}{N_\ell ^{{\texttt {cnt}}}+N_{{\texttt {shr}}}^{{\texttt {cnt}}}}. \end{aligned}$$

We multiply the sample size for cases (respectively, controls) in each study $\ell$ by $\lambda _\ell ^{{\texttt {cse}}}$ (respectively, $\lambda _\ell ^{{\texttt {cnt}}}$) before proceeding with the logistic regression described in “Logistic regression” section. See^51^ for a similar correction strategy. We finally note that in practice the exact number of overlapping samples between two studies is usually not known. In this case, we followed the approach proposed in^28^ to estimate the overlapping sample size.

#### Meta-analyzing multiple datasets

We now extend our approach to meta-analyze more than two datasets. The main difference with our previously described approach is the handling of the indicator variable for multiple datasets. We can still reconstruct the genotype count for each input study in exactly the same way as in Table 10 as well as the response vector following Eq. (4.1.3). Therefore, when multiple studies are meta-analyzed, ${\mathbf {g}}_i$ and ${\mathbf {y}}$ become

$$\begin{aligned} {\mathbf {g}}_i&= \left[ {\mathbf {g}}_{i1} \ldots {\mathbf {g}}_{iL}\right] ,\\ {\mathbf {y}}&= \left[ {\mathbf {y}}_{1} \ldots {\mathbf {y}}_{L}\right] . \end{aligned}$$

The indicator vector ${\mathbf {s}}$ cannot be binary anymore. Intuitively, one may consider using *L* binary vectors, each to encode samples from each input study. However, this approach would necessitate up to ${L(L-1)}/{2}$ vectors to encode pairwise sample overlap. This increases the computational complexity by $O(L^2)$. A simpler alternative is to use categorical variable as the source study indicator. Note that in this case, different rankings of the studies can lead to completely different results. A straightforward idea is to encode the studies using their population allele frequencies, which can be computed via Table 8 as follows:

$$\begin{aligned} I_{i\ell } = \frac{a_{i\ell }^{\text {cse}} + a_{i\ell }^{\text {cnt}}}{a_{i\ell }^{\text {cse}}+a_{i\ell }^{\text {cnt}}+u_{i\ell }^{\text {cse}}+u_{i\ell }^{\text {cnt}}} \end{aligned}$$

Note this is encoding also controls for population stratification across multiple sample sources. Then, when analyzing *L* studies, the indicator vector ${\mathbf {s}}$ becomes:

$$\begin{aligned} {\mathbf {s}} = \left[ \begin{array}{cc} \underbrace{I_1\ldots I_1}_{N_{1}^{\text {cse}}+N_{1}^{\text {cnt}}} \ldots \underbrace{I_L\ldots I_L}_{N_{L}^{\text {cse}}+N_{L}^{\text {cnt}}} \end{array}\right] . \end{aligned}$$

We can now proceed with the logistic regression as in “Logistic regression” section. In order to handle sample overlap across multiple studies, we use the subscript $(\cdot )_{\ell _1 \ell _2}$ to denote properties of shared samples between two studies $\ell _1$ and $\ell _2$. Then, generalizing Eqs. (9) and (10), we get, for each pair of input studies $\ell _1$ and $\ell _2$,

$$\begin{aligned} N_{\ell _1\ell _2}^{{\texttt {cse}}}&= N_{\ell _1\ell _2}^{{\texttt {cse-cse}}} + \frac{N_{\ell _1\ell _2}^{{\texttt {cse-cnt}}}+N_{\ell _1\ell _2}^{{\texttt {cnt-cse}}}}{2},\\ N_{\ell _1\ell _2}^{{\texttt {cnt}}}&= N_{\ell _1\ell _2}^{{\texttt {cnt-cnt}}} + \frac{N_{\ell _1\ell _2}^{{\texttt {cnt-cse}}}+N_{\ell _1\ell _2}^{{\texttt {cse-cnt}}}}{2}. \end{aligned}$$

Finally, for any study $\ell _1 =1 \ldots L$, the sample size correction is

$$\begin{aligned} \lambda _{\ell _1}^{{\texttt {cse}}}&= \frac{N_{\ell _1}^{{\texttt {cse}}}}{N_{\ell _1}^{{\texttt {cse}}}+ \sum _{\ell _2 \ne \ell _1}^L N_{\ell _1\ell _2}^{{\texttt {cse}}}},\\ \lambda _{\ell _1}^{{\texttt {cnt}}}&= \frac{N_{\ell _1}^{{\texttt {cnt}}}}{N_{\ell _1}^{{\texttt {cnt}}}+\sum _{\ell _2 \ne \ell _1}^L N_{\ell _1\ell _2}^{{\texttt {cnt}}}}. \end{aligned}$$

We can now apply $\lambda _{\ell _1}^{{\texttt {cse}}}$ to correct the sample size for cases in study $\ell _1$ and we can apply $\lambda _{\ell _1}^{{\texttt {cnt}}}$ to correct the sample size for controls and proceed with logistic regression.

### cc-GWAS and group PRS

#### cc-GWAS using summary statistics

cc-GWAS is a straight-forward approach to investigate the genetic differences between two traits. However, in practice, it is usually challenging and time consuming, due to restrictions in individual level data sharing. Recently, a method for cc-GWAS that relies only on summary statistics has been proposed in^16^. We propose an alternative perspective on summary-statistics-based cc-GWAS framework, using the foundations of “Reconstructing allele counts” section.

One of the biggest challenges of cc-GWAS is the differentiation of the genetic effects from trait-trait difference and population stratification. Assume that for a fixed SNP, we run logistic regression focusing only on the cases of the two studies. Let ${\mathbf {y}}^{{\texttt {cse}}}_j = 1$ denote that sample *j* is a case from the first study and let ${\mathbf {y}}^{{\texttt {cse}}}_j = 0$ denote that *j* is a case from the second study. Let ${\mathbf {g}}^{{\texttt {cse}}}_j$ be the genotype of the *j*-th case. Then,

11 $$\begin{aligned} {{\mathsf {Pr}}}({\mathbf {y}}_j^{{\texttt {cse}}} = 1 | {\mathbf {g}}^{{\texttt {cse}}}_j) = S(\beta _0^{{\texttt {cse}}} + \beta _1^{{\texttt {cse}}} {\mathbf {g}}^{{\texttt {cse}}}_j). \end{aligned}$$

The effect size $\beta _1^{{\texttt {cse}}}$ that is the output of logistic regression will include effects from the real genetic differences between trait 1 and trait 2 ($\beta _g$) as well as from population stratification ($\beta _s$). We can assume that these two effects are independent of each other:

$$\begin{aligned} \beta _1^{{\texttt {cse}}} = \beta _g + \beta _s. \end{aligned}$$

Assume that the control samples from studies one and two *do not carry the traits of interest*. Then, we can estimate the effect of population stratification by running another logistic regression, focusing only on controls from the two studies, as follows:

12 $$\begin{aligned} {{\mathsf {Pr}}}({\mathbf {y}}_j^{{\texttt {cnt}}} = 1 | {\mathbf {g}}^{{\texttt {cnt}}}_j) = S(\beta _0^{{\texttt {cnt}}} + \beta _s {\mathbf {g}}^{{\texttt {cnt}}}_j). \end{aligned}$$

In the above, ${\mathbf {y}}^{{\texttt {cnt}}}_j = 1$ denotes that sample *j* is a control from study one, ${\mathbf {y}}^{{\texttt {cnt}}}_j = 0)$ denotes that *j* is a control from study two, and ${\mathbf {g}}^{{\texttt {cnt}}}_j$ denotes the the genotype for the *j*-th control sample. From this logistic regression, we can get an estimate of the stratification effect $\beta _s$. Note that along with $\beta _s$, we will also get a standard error for the estimate of stratification ${{\mathrm {SE}}}_s$, which essentially corresponds to the sample size of controls in the two input studies. If we do not have a good amount of controls, ${{\mathrm {SE}}}_s$ will turn out to be large, indicating that the estimate for stratification effect is not reliable and the results from the cc-GWAS should be interpreted carefully.

If ${{\mathrm {SE}}}_s$ is small enough, then it is reasonable to assume that the estimate of the stratification effect is credible and we can subsequently treat $\beta _s$ as a fixed value. Then, the genetic effect from the trait-trait difference that we are interested in is

13 $$\begin{aligned} \beta _g = \beta _1^{{\texttt {cse}}} - \beta _s. \end{aligned}$$

It now follows that the standard error of $\beta _g$ is

14 $$\begin{aligned} {{\mathrm {Var}}}(\beta _g) = {{\mathrm {Var}}}(\beta _1^{{\texttt {cse}}}) \implies {{\mathrm {SE}}}_{g} = {{\mathrm {SE}}}_1, \end{aligned}$$

using the derivations of “Reconstructing genotype counts” section. Logistic regressions on cases (Eqs. (11)) and controls (Eq. (12)) can be carried out as discussed in “Logistic regression” section, with minor changes (include only the designated samples; relabel the dependent variable; and remove the indicator variable). By running these two logistic regressions, we can compute $\beta _1^{{\texttt {cse}}}, \beta _s, SE_1^{{\texttt {cse}}}$, and $SE_s$. Then, using Eqs. (13) and (14), we can compute $\beta _g$ and $SE_g$ for each SNP. Similarly, we can also compute the corresponding *p*-value using a *Z*-distribution for $\left| \frac{\beta _g}{SE_g}\right|$.

#### Mean PRS for cases and controls

Recall that the PRS for the *t*-th individual in the study is computed as:

15 $$\begin{aligned} \text {PRS}_{t} = \sum _{i=1}^M \frac{S_i \cdot g_{it}}{2M}, \end{aligned}$$

where $g_{it}$ is the genotype of the *i*-th SNP for the *t*-th individual and $S_i$ is the weight for SNP *i*, which is usually defined as

$$\begin{aligned} S_i = \log (\text {OR}_i^{{\texttt {base}}}), \end{aligned}$$

where $\text {OR}_i^{{\texttt {base}}}$ is the odds ratio of SNP *i* in the base summary statistics. Recall from “Notation” section that *M* is the total number of SNPs. Then, in order to compute the average PRS for, say, cases, we simply need to sum up the individual PRS and average over the number of cases. More precisely,

$$\begin{aligned} \text {PRS}^{{\texttt {cse}}} = \frac{1}{2 M N^{{\texttt {cse}}}}\sum _{t \in {\texttt {cse}}} \sum _{i=1}^M S_i \cdot g_{it}. \end{aligned}$$

where $N^{{\texttt {cse}}}$ is the number of cases in the target study. The above equation can be rewritten as

$$\begin{aligned} \text {PRS}^{{\texttt {cse}}} = \frac{1}{2 M N^{{\texttt {cse}}}}\sum _{i=1}^M S_i \sum _{t \in {\texttt {cse}}} g_{it}. \end{aligned}$$

Notice that in an additive model, ${\sum _{t \in {\texttt {cse}}} g_{it}}/{2N^{\texttt {cse}}}$ is the allele frequency of SNP *i* over all cases in the target study, which can be computed using only the summary statistics as shown in “Reconstructing genotype counts” section and Table 9. Thus, the mean PRS under an additive model for cases and controls can be computed as follows:

$$\begin{aligned} \text {PRS}^{{\texttt {cse}}}&= \frac{\sum _{i=1}^M S_i p_{i}^{{\texttt {cse}}}}{M},\\ \text {PRS}^{{\texttt {cnt}}}&= \frac{\sum _{i=1}^M S_i p_{i}^{{\texttt {cnt}}}}{M}. \end{aligned}$$

All relevant information for this computation can be easily obtained from the summary statistics of the base and/or target study.

#### Estimating the standard deviation of the PRS for cases and controls

Interestingly, we can also estimate the standard deviation of the PRS for cases and controls, even without individual level genotype information, under mild assumptions. First, from Eq. (15), we compute the variance of an individual’s PRS as follows:

16 $$\begin{aligned} {{\mathrm {Var}}}(\text {PRS}_{t})&= {{\mathrm {Var}}}(\sum _{i=1}^M \frac{S_i \cdot g_{it}}{2M}) \nonumber \\&= \frac{1}{4M^2}{{\mathrm {Var}}}(\sum _{i=1}^M S_i \cdot g_{it}). \end{aligned}$$

Recall that as a general step prior to the computation of PRS, it is recommended to prune or clump the SNPs used for the PRS computation. Therefore, our first assumption is that the $g_{it}$’s are pairwise independent. Then, Eq. (16) can be simplified as follows:

17 $$\begin{aligned} {{\mathrm {Var}}}(\text {PRS}_{t})&= \frac{\sum _{i=1}^M {{\mathrm {Var}}}(S_i \cdot g_{it})}{4M^2} \nonumber \\&= \frac{\sum _{i=1}^M S_i^2 {{\mathrm {Var}}}(g_{it})}{4M^2}. \end{aligned}$$

Notice that under an additive model, $g_{it}$ is a discrete random variable that only takes the value zero, one, and two. Consider all cases and, as in “Reconstructing genotype counts” section, assume that the SNPs are in HWE. Then, the distribution of $g_{it}$ in the cases is presented in Table 12.

**Table 12:** The probability distribution of $g_{it}$ for SNP *i*.

$g_{it} = 2$ (two copies of $A_1$)	$g_{it} = 1$ (one copy of $A_1$)	$g_{it} = 0$ (zero copies of $A_1$)
$(p^{{\texttt {cse}}}_i)^2$	$2p^{{\texttt {cse}}}_iq^{{\texttt {cse}}}_i$	$(q^{{\texttt {cse}}}_i)^2$

In this table, $p^{{\texttt {cse}}}_i$ denotes the allele frequency of $A_1$ in cases and $q^{{\texttt {cse}}}_i = 1-p^{{\texttt {cse}}}_i$.

We can now compute the variance of $g_{it}$ in cases as follows:

$$\begin{aligned} {{\mathrm {Var}}}(g_{it})&= {{\mathrm {E}}}(g_{it}^2) - ({{\mathrm {E}}}g_{it})^2 \\&= (2p^{{\texttt {cse}}}_iq^{{\texttt {cse}}}_i+4(p^{{\texttt {cse}}}_i)^2) - (2p^{{\texttt {cse}}}_iq^{{\texttt {cse}}}_i+2(p^{{\texttt {cse}}}_i)^2)^2 \\&= (2p^{{\texttt {cse}}}_iq^{{\texttt {cse}}}_i+4(p^{{\texttt {cse}}}_i)^2) - (2p^{{\texttt {cse}}}_i(p^{{\texttt {cse}}}_i+q^{{\texttt {cse}}}_i))^2 \\&= 2p^{{\texttt {cse}}}_iq^{{\texttt {cse}}}_i+4(p^{{\texttt {cse}}}_i)^2 - 4(p^{{\texttt {cse}}}_i)^2 = 2p^{{\texttt {cse}}}_iq^{{\texttt {cse}}}_i. \end{aligned}$$

Substituting into Eq. (17), we get

$$\begin{aligned} {{\mathrm {Var}}}(\text {PRS}^{{\texttt {cse}}})&= \frac{\sum _{i=1}^M S_i^2( 2p^{{\texttt {cse}}}_iq^{{\texttt {cse}}}_i)}{4M^2}. \end{aligned}$$

Similarly, we can compute the estimated variance $\text {PRS}^{{\texttt {cnt}}}$ for controls and $\text {PRS}$ for the overall population of the target study. To summarize, our estimates are

$$\begin{aligned} {{\mathrm {Var}}}(\text {PRS}^{{\texttt {cse}}})&= \frac{\sum _{i=1}^M S_i^2 p_i^{{\texttt {cse}}}q_i^{{\texttt {cse}}}}{2M^2},\\ {{\mathrm {Var}}}(\text {PRS}^{{\texttt {cnt}}})&= \frac{\sum _{i=1}^M S_i^2 p_i^{{\texttt {cnt}}}q_i^{{\texttt {cnt}}}}{2M^2},\\ {{\mathrm {Var}}}(\text {PRS})&= \frac{\sum _{i=1}^M S_i^2 p_iq_i}{2M^2}. \end{aligned}$$

Here $p_i$ is the frequency of allele $A_1$ for SNP *i* in all samples of the target study, and can be computed as:

$$\begin{aligned} p_i&= \frac{N^{{\texttt {cse}}} p_i^{{\texttt {cse}}} + N^{{\texttt {cnt}}}p_i^{{\texttt {cnt}}}}{N^{{\texttt {cse}}}+N^{{\texttt {cnt}}}}, \\ q_i&= 1-p_i. \end{aligned}$$

We can now apply a *t*-test in order to obtain a *p*-value for the difference between the PRS distributions in cases and controls. Given the estimated group means and standard deviations for cases and controls, we can further assume that the individual level PRS follow a normal distribution in each group and use the *t*-test statistic as follows:

$$\begin{aligned} t = \frac{\text {PRS}^{{\texttt {cse}}}-\text {PRS}^{{\texttt {cnt}}}}{\sqrt{{{\mathrm {Var}}}(\text {PRS})} \cdot \sqrt{\frac{1}{N^{{\texttt {cse}}}} + \frac{1}{N^{{\texttt {cnt}}}}}}. \end{aligned}$$

Finally, the degrees of freedom are given by $df = N^{{\texttt {cse}}} + N^{{\texttt {cnt}}} - 2$.

### Experiments

#### Data

Synthetic data. We used the Balding-Nichols model^26,52^ for synthetic genotype generation, assuming a minor allele frequency (MAF) of 0.3 for each SNPs and a relative risk r (*r* = 1.15/1.2/1.3) for the effective alleles of the causal SNPs in each population. The simulation was carried out under a range of $F_{st}$ values ($F_{st} = 0.01/0.05/0.1$). For the fixed-effect meta-analysis, we simulated 1000 cases and 1000 controls for each input study. A total of 100,000 SNPs were generated, out of which 1000 are causal SNPs with the predefined risk for the effective alleles. Moreover, on top of the independent populations, we also evaluated the performance of ReACt under the presence of sample overlap by introducing a predefined amount of samples shared between each pair of input studies (100 cases, 100 controls overlap; or 500 cases, 500 controls overlap).

To further demonstrate the scalability of ReACt, we evaluated its performance on UK biobank samples with phenotypes simulated using the gcta tool^29^. The simulation was carried out using quality controlled genotypes (removing SNPs and individuals showing missing rate larger than 0.02 and SNPs strongly violating the Hardy-Weinberg equilibrium with a *p*-value larger than $10^{-6}$), using a predefined trait heritability equal to 0.4 and prevalence equal to 0.2. We simulated 50,000 cases and 250,000 controls, each genotyped on 634,758 SNPs, out of which 1000 SNPs were randomly selected to be causal with effect size *OR* equal to 1.2. In each iteration, we split the samples into two equal sized subsets, each with 25,000 cases and 125,000 controls. Similarly to our experiments on the Balding-Nichols model, we tested the performance under various degrees of sample overlap.

For the cc-GWAS, inspired by^16^, we used the same simulation model but introduced three types of SNPs for a thorough evaluation of the method’s robustness: *(i)* SNPs with non-zero effect in only one of the studies and zero effect in the other; *(ii)* SNPs with zero effect in both input studies; and *(iii)* SNPs with the same non-zero effect size (predefined *r*) in both input studies. All of the three types of SNPs would suffer from population stratification at a predefined value of $F_{st}$. In total, 100,000 SNPs were generated, with 1000 (for each input study) from type (i), 49,000 from type (ii), and 49,000 from type (iii). To investigate the effect of study sizes, we evaluated the method performance on input studies with 2000 cases and 2000 controls each, as well as on studies with 5000 cases and 5000 controls each.

Individual level genotype data. We tested the performance of our fixed-effect meta-analysis method and group PRS method on the depressive episode trait in UK biobank dataset^30^. Only independent European ancestry samples identified through PCA and IBD check are included for the analysis. We applied basic quality control filters on those samples, which were removing SNPs and samples with a missing rate exceeding $2\%$ or violating the Hardy-Weinberg equilibrium ($p_{HWE} < 10^{-6}$). As a result, 640,756 SNPs and 331,217 samples (18,368 cases and 312,849 controls) survived and were used for the experiment. For the evaluation of the fixed-effect meta-analysis method, we ran a standard GWAS with all samples and treated SNPs with $p < 10^{-6}$ from the results as the “true signals” to be captured. For all GWAS on UB biobank samples, we correct for age, gender, sample collection batch and top 10 PCs obtained using software TeraPCA^53^.

Generating summary statistics. For synthetic data and individual level genotypes, summary statistics were generated using PLink^47^, correcting for the top ten principal components (PCs) in the case of admixed datasets. For real individual level genotype data, we divided the samples randomly into two equal sized subsets and ran a GWAS on each subset separately to obtain summary statistics for each subset. We performed ten such random iterations in our experimental evaluations. For the fixed-effect meta-analysis, on top of two independent subsets, we also introduced 100/500 sample overlap for synthetic data under the Balding-Nichols model; 5000/10,000 sample overlap for synthetic data under the gcta model; and 500/1000 sample overlap for the real GWAS data on depression.

Publicly available summary statistics. As part of the performance evaluation for our group PRS method, we used summary statistics from an MDD GWAS published in 2013^36^ as the base study. Most recent large-scale GWAS often include UK biobank as part of the samples. We chose to use an earlier GWAS published *before* the release of UK biobank data in order to minimize sample overlap between the base and target populations as much as possible. The summary statistics contains in total 1,235,109 SNPs on genome build hg18. After liftover^54^ to hg19, 1,234,855 remained for the analysis.

For group PRS and cc-GWAS, we demonstrated the applicability of our methods using publicly available summary statistics. We chose the summary statistics of eight neuropsychiatric disorders made available by the Psychiatric Genomics Consortium (PGC), since the underlying relationships between this set of disorders has been relatively well-studied. Information on the eight summary statistics can be found in Table 13.

**Table 13:** Information on summary statistics for the eight psychiatric disorders used in the experiments.

Disorder	#Cases	#Controls	Total	#SNPs	Reference
Obsessive-compulsive disorder (OCD)	2688	7037	9725	8,409,516	^55^
Tourette syndrome (TS)	4819	9488	14,307	8,947,432	^56^
Eating disorder (ED)	3495	10,982	14,477	10,641,224	^57^
Autism spectrum disorder (ASD)	18,382	27,969	46,351	9,112,386	^58^
Bipolar disorder (BIP)	20,352	31,358	51,710	13,413,244	^32^
Schizophrenia (SCZ)	36,989	113,075	150,064	9,075,843	^33^
Attention-deficit/hyperactivity disorder (ADHD)	19,099	34,194	53,293	8,094,094	^59^
Major depression (MD)	69,232	161,009	230,241	9,874,289	^60^

Note that we used summary statistics only for samples of European ancestry. For MD, we used the summary statistics generated by UK biobank, excluding the 23andMe samples; for BIP, we used the summary statistics including all three patient sub-types.

#### Evaluation metrics

Fixed-effect meta-analysis. For synthetic experiments using the Balding-Nichols model where all SNPs were simulated independently, results after performing the meta-analysis were compared with the predefined causal variants. Under each experimental condition, we reported the average true positive rate (i.e., the percentage of predefined causal SNPs identified under the designated significant threshold), as well as the false positive rate (type I error, i.e., the percentage of non-causal SNPs falsely identified as causal under the same significance threshold) out of ten independent iterations.

For experiments under the gcta simulator it was unreasonable to report power and type I error rates by comparing with the predefined causal SNPs, since the SNPs were not independent in the input genotypes. Therefore, for this experiment, the performance of ReACt and the other tools was evaluated by comparing results to the outcome of a GWAS on all 50,000 cases and 250,000 controls, where 1,886 SNPs were identified as genome-wide significant (GWAS *p*-value $< 5\times 10^{-8}$). We considered those 1,886 SNPs as true signals (“causal SNPs”) and reported average power and type I error rates over ten iterations for all methods.

For real genotype data, in each iteration, we meta-analyzed summary statistics of two subsets using the proposed methods and standard approaches and compared results with the GWAS results on the complete dataset. Following the lines of the experiments using the gcta simulation model, we again reported results averaged over ten iterations (random splits) showing, on average, how many times a SNP reported as a “true signal” in the overall GWAS got picked up by each meta-analysis method (true positive) as well as how many extra SNPs each method identified (false positive). The performance on real genotype data was also evaluated under 0/100/500 sample overlap. Sample size for each subset under different conditions was 482 cases, 993 controls with no sample overlap; 532 cases, 1043 controls with 100 cases and 100 controls overlap; and 732 cases, 1243 controls with 500 cases and 500 controls overlap.

We compared the performance of ReACt in terms of accuracy as well as running time with METAL^24^ and ASSET^25^, which are both widely used tools for fixed-effect meta-analysis. Note that the latest stable release of METAL does not have the sample overlap correction functionality implemented. Therefore, for performance comparison, we used the *development version* available on GitHub^28^.

cc-GWAS. Out of the three types of SNPs generated for the cc-GWAS evaluation (see “Data” section), we expect ReACt to pick up only type (i) SNPs as they have been designed to be the trait differential SNPs. Therefore, we reported the power (i.e., the percentage of type (i) SNPs identified under the significance threshold) of ReACt based on the number of type (i) SNPs that were identified as well as type I error rates (i.e., the percentage of type (ii) or (iii) SNPs falsely picked up under the same significance threshold) for type (ii) SNPs and type (iii) SNPs. Since the randomness introduced by the simulation could lead to false positives that were not due to the method itself, we filtered out type (iii) SNPs showing extreme differences in effect size between studies, by removing type (iii) SNPs with $|OR_{i1} - OR_{i2}| \ge 0.1$ from performance evaluation. Here $OR_{i1}$ corresponds to the odd ratio for the *i*th SNP in the first study and $OR_{i2}$ corresponds to the odd ratio for the *i*th SNP in the other study. Since all three types of SNPs suffered from population stratification, we evaluated the performance of ReACt under a challenging scenario. Besides simulation, experiments using summary statistics for schizophrenia (SCZ)^33^ and bipolar disorder (BIP)^32^ were also carried out. These two disorders were chosen due to the existence of case-case association study using the individual level genotypes^34^. We tested ReACt using the summary statistics and compared the results with the existing case-case association study between SCZ and BIP to see whether it could detect possible genetic differences between the two disorders. Since no individual level quality control could be carried out, we expected our results to correspond to a case-case GWAS including 36,989 cases from SCZ and 20,352 cases from all three sub-types of BIP (type 1, type 2, and schizoaffective bipolar disorder). SNPs on the X-chromosome were excluded from this analysis. Further, to make our protocol comparable to the ones used in^31^, we also removed variants on the MHC region (chr6: 25,000,000–35,000,000BP). From a theoretical perspective, our approach analyzes each SNP independently. Therefore, removing MHC is not mandatory to run ccGWAS using ReACt, unless the study design requires to do so. As a result, a total of 9,018,199 SNPs shared between both summary statistics were used for the analysis. The results were compared in detail with the results reported by the cc-GWAS in^16^.

Group PRS. In order to show that our method outputs reliable estimates of the group-wise statistics for PRS without accessing individual level genotypes, we compared the output of our method to the true group mean and standard deviation computed from the individual level PRS on synthetic data, as described in “Data” section. Performance was evaluated under with a fixed 0.05 $F_{st}$ between the base and target studies. For a pair of base and target studies , we estimated the mean PRS for case/control groups as well as their standard deviation using SNPs with *p*-values strictly less than $5 \cdot 10^{-5}$ in the summary statistics. We also computed the individual level PRS using PRSise2 to obtain the true group mean and standard deviation. Our experiments show that our estimates are numerically close to the real values. Next, we evaluated the performance of ReACt on real GWAS datasets, where the individual level genotype of the target study was available. For this experiment, we used an earlier GWAS summary statistics of MDD^36^ as the base study (see “Data” section for details) and cases and matching controls of depressive episode trait in UK biobank as the target population^30^. We clumped the base summary statistics using the European samples from 1000 Genome Project as reference, under parameters –clump-p1 1 –clump-kb 250 –clump-r2 0.1. We tested the method and reported results under a range of *p*-value thresholds ($0.1, 0.01, 0.001, 10^{-4}$). For each threshold, we used only independent SNPs with a *p*-value smaller than the respective threshold from the base summary statistics for PRS calculation, using both ReACt and PRSice2 ^35^. We reported the mean PRS of cases and controls, as well as the resulting *p*-value from *t*-test. In the case of PRSice2, we also reported the regression $r^2$ value and *p*-value for the PRS predictor with and without correcting for covariates (ie., the top five principal components).

Finally we applied ReACt to summary statistics of eight neuropsychiatric disorders (OCD, TS, ED, ADHD, ASD, BIP, SCZ and MDD, see “Data” section for details) and reported the pairwise PRS prediction power in terms of *t*-test *p*-values for the difference between case/control group PRS means. Prior to the group PRS computation, each base summary statistics was clumped using PLink^47^ using parameters –clump-p1 1 –clump-kb 250 –clump-r2 0.1, with the European samples from 1000 Genome Project as a reference. All PRS values were estimated using independent SNPs with *p*-values strictly less than $10^{-5}$ from the base summary statistics.

## Supplementary Information

Supplementary Information 1.

Supplementary Information 2.

## Data Availability

Summary statistics for the eight disorders used in this study can be downloaded from Psychiatric Genomics Consortium (PGC): https://www.med.unc.edu/pgc/download-results/. In-house script used for synthetic data generation can be found from our github page https://github.com/Paschou-Lab/ReAct/tree/main/Simulator. Some data that support the findings of this study are available from the UK biobank but restrictions apply to the availability of these data, which were used under license for the current study, and so are not publicly available. Data are however available from the authors upon reasonable request and with permission of UK biobank. This research has been conducted using the UK Biobank Resource under Application Number 61553. An implementation for ReACt can be found on our github page: https://github.com/Paschou-Lab/ReACt.
