# Supplementary material for: Reconstructing SNP allele and genotype frequencies from GWAS summary statistics
Source: Sci Rep. 2022 May 17;12:8242. doi: 10.1038/s41598-022-12185-6 (PMC9114146; doi:10.1038/s41598-022-12185-6)
Supplement: Supplementary file 1 — Supplementary Information 1. [file 41598_2022_12185_MOESM1_ESM.pdf]

5 Supplementary Material

5.1 Supplementary tables

Table S1. Average running time in seconds for fixed effect meta-analysis for ReACt, METAL, and ASSET. All experiments were performed at Purdue's Snyder cluster on a dedicated node which features a Haswell processor running at 2.6 GHz with 512 GB of RAM and a 64-bit CentOS Linux 7 operating system. We report average running time in seconds over ten iterations using `ReACt`, `METAL`, and `ASSET`. In the case of METAL we evaluated the performance of the latest release in GitHub [28]. In each iteration, two or four sets of summary statistics (for 100,000 SNPs) were meta-analyzed. Recall that all methods scale as a function of the number of SNPs and is independent of the number of samples, since only summary statistics are used.

	`ReACt`	`METAL`	`ASSET`
2 input studies	2.2s	1.8s	696s
4 input studies	3.1s	3.3s	3715s

Table S2. Performance of fixed-effect meta-analysis with two input studies with uneven case/control sample sizes under different conditions. We compare power and type I error rate (T1E) of our method meta-analyzing two studies with uneven case/control sample sizes vs. ASSET/METAL for a significance threshold $p < 5 \cdot 10^{-5}$. Study one contains 1500 cases and 500 controls, and study two contains 500 cases and 1500 controls.

risk	Fst	`ReACt`		`METAL/ASSET`	
		Power	T1E	Power	T1E
1.15	0.01	4.89E-02	4.24E-05	4.97E-02	4.65E-05
	0.05	5.07E-02	4.65E-05	5.13E-02	4.95E-05
	0.1	4.35E-02	4.04E-05	4.37E-02	4.55E-05
1.2	0.01	1.79E-01	4.75E-05	1.80E-01	5.05E-05
	0.05	1.66E-01	6.36E-05	1.67E-01	6.77E-05
	0.1	1.64E-01	4.44E-05	1.65E-01	4.55E-05
1.3	0.01	6.28E-01	4.24E-05	6.30E-01	4.44E-05
	0.05	5.99E-01	4.85E-05	6.00E-01	4.55E-05
	0.1	5.63E-01	4.65E-05	5.64E-01	4.85E-05

Table S3. Performance of fixed-effect meta-analysis with two input studies under different conditions. We compare power and type I error rate (T1E) of our method meta-analyzing two studies vs. ASSET/METAL for a significance threshold $p < 5 \cdot 10^{-5}$. METAL dev refers to the latest release in GitHub [28]. Two variants of ReACt are tested: Exact and Est, indicating whether the sample overlap was *exactly* known as part of the input or whether it was *estimated*, respectively. Sample overlap indicates the number of cases and controls that were shared between two input studies. I.e. a sample overlap equal to 100 means that there are 100 cases **and** 100 controls shared between two input studies. Total sample sizes for each input study, including the shared samples, are equal to 2000 when the sample overlap is equal to zero; 2400 when the sample overlap is equal to 100; and 4000 when the sample overlap is equal to 500. In each case, the sample is equally split to cases and controls. Also see figure 1 and 2.

risk	Fst	overlap	ASSET		ReACt (Exact)		ReACt (Est.)		METAL (dev)	
			Power	T1E	Power	T1E	Power	T1E	Power	T1E
1.15	0.01	0	1.04E-01	4.95E-05	1.03E-01	4.85E-05	-	-	1.04E-01	4.95E-05
		100	1.13E-01	4.34E-05	1.27E-01	5.25E-05	1.30E-01	4.85E-05	1.31E-01	5.15E-05
		500	1.69E-01	1.11E-05	2.79E-01	4.75E-05	2.80E-01	4.85E-05	2.80E-01	4.65E-05
	0.05	0	9.66E-02	5.25E-05	9.31E-02	5.25E-05	-	-	9.66E-02	5.25E-05
		100	9.68E-02	3.43E-05	1.19E-01	4.14E-05	1.17E-01	4.14E-05	1.17E-01	4.65E-05
		500	1.53E-01	4.04E-06	2.68E-01	3.84E-05	2.69E-01	3.74E-05	2.67E-01	3.74E-05
	0.1	0	8.65E-02	4.34E-05	8.19E-02	4.04E-05	-	-	8.65E-02	4.34E-05
		100	7.75E-02	3.33E-05	1.05E-01	4.44E-05	1.09E-01	4.65E-05	1.08E-01	5.15E-05
		500	1.24E-01	9.09E-06	2.39E-01	4.65E-05	2.42E-01	4.95E-05	2.41E-01	5.15E-05
1.2	0.01	0	3.21E-01	3.84E-05	3.18E-01	3.74E-05	-	-	3.21E-01	3.84E-05
		100	3.41E-01	3.54E-05	3.82E-01	4.04E-05	3.85E-01	4.04E-05	3.85E-01	4.14E-05
		500	4.95E-01	7.07E-06	6.44E-01	4.04E-05	6.47E-01	4.24E-05	6.46E-01	4.14E-05
	0.05	0	3.13E-01	4.24E-05	3.06E-01	3.94E-05	-	-	3.13E-01	4.24E-05
		100	2.96E-01	4.65E-05	3.59E-01	5.35E-05	3.66E-01	5.35E-05	3.65E-01	5.76E-05
		500	4.47E-01	8.08E-06	6.09E-01	4.85E-05	6.14E-01	5.15E-05	6.11E-01	5.25E-05
	0.1	0	2.83E-01	4.85E-05	2.71E-01	4.44E-05	-	-	2.83E-01	4.85E-05
		100	2.45E-01	4.44E-05	3.28E-01	4.34E-05	3.27E-01	4.55E-05	3.23E-01	4.55E-05
		500	3.95E-01	8.08E-06	5.76E-01	4.75E-05	5.83E-01	4.85E-05	5.80E-01	4.65E-05
1.3	0.01	0	8.00E-01	3.23E-05	7.99E-01	3.23E-05	-	-	8.00E-01	3.23E-05
		100	6.80E-01	3.84E-05	7.36E-01	4.65E-05	7.43E-01	5.15E-05	7.42E-01	5.45E-05
		500	4.90E-01	4.04E-06	6.40E-01	2.42E-05	6.98E-01	5.35E-05	6.97E-01	5.05E-05
	0.05	0	7.82E-01	4.95E-05	7.77E-01	4.44E-05	-	-	7.82E-01	4.95E-05
		100	6.32E-01	3.94E-05	7.48E-01	4.55E-05	7.55E-01	5.25E-05	7.52E-01	5.45E-05
		500	4.99E-01	1.01E-06	6.67E-01	1.31E-05	7.18E-01	4.04E-05	7.16E-01	3.64E-05
	0.1	0	7.32E-01	4.95E-05	7.20E-01	4.44E-05	-	-	7.32E-01	4.95E-05
		100	6.01E-01	3.84E-05	7.67E-01	4.24E-05	7.71E-01	4.65E-05	7.62E-01	5.15E-05
		500	5.49E-01	1.01E-06	7.30E-01	1.31E-05	7.67E-01	3.43E-05	7.63E-01	3.94E-05

Table S4. Performance of fixed-effect meta-analysis with four input studies under different conditions. We compare power and type I error rate (T1E) of our method meta-analyzing four studies vs. ASSET/METAL for a significance threshold $p < 5 \cdot 10^{-5}$. METAL dev refers to the latest release in GitHub [28]. Two variants of ReACt are tested: Exact and Est, indicating whether the sample overlap was *exactly* known as part of the input or whether it was *estimated*, respectively. Sample overlap indicates the number of cases and controls that were shared between two input studies. I.e. a sample overlap equal to 100 means that there are 100 cases **and** 100 controls shared between two input studies. Total sample sizes for each input study, including the shared samples, are equal to 2000 when the sample overlap is equal to zero; 2400 when the sample overlap is equal to 100; and 4000 when the sample overlap is equal to 500. In each case, the sample is equally split to cases and controls.

risk	Fst	overlap	ASSET		ReACt (Exact)		ReACt (Est.)		METAL (dev)	
			Power	T1E	Power	T1E	Power	T1E	Power	T1E
1.15	0.01	0	4.31E-01	4.75E-05	4.31E-01	4.75E-05	-	-	4.31E-01	4.75E-05
		100	3.19E-01	2.93E-05	4.00E-01	5.15E-05	4.03E-01	5.45E-05	4.03E-01	4.85E-05
		500	2.36E-01	1.01E-06	5.20E-01	4.85E-05	5.27E-01	5.25E-05	5.23E-01	4.85E-05
	0.05	0	4.13E-01	4.34E-05	4.08E-01	4.24E-05	-	-	4.13E-01	4.34E-05
		100	2.49E-01	3.33E-05	3.83E-01	5.25E-05	3.85E-01	5.66E-05	3.78E-01	5.56E-05
		500	2.06E-01	2.02E-06	5.03E-01	5.56E-05	5.14E-01	6.46E-05	5.04E-01	5.25E-05
	0.1	0	3.72E-01	5.35E-05	3.64E-01	4.85E-05	-	-	3.72E-01	5.35E-05
		100	1.90E-01	2.42E-05	3.46E-01	4.55E-05	3.53E-01	5.66E-05	3.41E-01	5.45E-05
		500	1.60E-01	2.02E-06	4.56E-01	5.15E-05	4.66E-01	5.45E-05	4.61E-01	5.35E-05
1.2	0.01	0	7.87E-01	5.15E-05	7.85E-01	5.15E-05	-	-	7.87E-01	5.15E-05
		100	6.48E-01	4.14E-05	7.59E-01	4.85E-05	7.64E-01	5.45E-05	7.59E-01	4.95E-05
		500	6.14E-01	0.00E+00	8.43E-01	5.05E-05	8.49E-01	5.96E-05	8.48E-01	5.25E-05
	0.05	0	7.61E-01	3.43E-05	7.57E-01	3.23E-05	-	-	7.61E-01	3.43E-05
		100	5.26E-01	1.82E-05	7.32E-01	3.54E-05	7.41E-01	4.85E-05	7.33E-01	4.65E-05
		500	5.36E-01	1.01E-06	8.19E-01	2.93E-05	8.28E-01	3.54E-05	8.23E-01	3.23E-05
	0.1	0	7.21E-01	5.15E-05	7.11E-01	5.15E-05	-	-	7.21E-01	5.15E-05
		100	4.22E-01	3.43E-05	6.88E-01	5.35E-05	6.86E-01	5.15E-05	6.76E-01	6.16E-05
		500	4.65E-01	1.01E-06	7.86E-01	4.65E-05	7.91E-01	5.25E-05	7.88E-01	5.15E-05
1.3	0.01	0	9.83E-01	5.45E-05	9.83E-01	5.45E-05	-	-	9.83E-01	5.45E-05
		100	8.59E-01	2.02E-05	9.45E-01	3.23E-05	9.54E-01	4.95E-05	9.50E-01	4.85E-05
		500	6.30E-01	0.00E+00	8.53E-01	5.05E-06	9.12E-01	6.46E-05	9.10E-01	6.87E-05
	0.05	0	9.71E-01	4.65E-05	9.70E-01	4.44E-05	-	-	9.71E-01	4.65E-05
		100	7.68E-01	2.22E-05	9.49E-01	3.23E-05	9.55E-01	5.15E-05	9.50E-01	4.85E-05
		500	6.10E-01	0.00E+00	8.73E-01	1.01E-05	9.23E-01	7.07E-05	9.21E-01	6.67E-05
	0.1	0	9.54E-01	5.66E-05	9.52E-01	4.65E-05	-	-	9.54E-01	5.66E-05
		100	6.91E-01	2.32E-05	9.45E-01	4.04E-05	9.47E-01	4.65E-05	9.40E-01	5.15E-05
		500	6.21E-01	0.00E+00	8.93E-01	1.01E-05	9.27E-01	4.04E-05	9.24E-01	4.55E-05

Table S5. Performance of sample overlap correction for estimating PRS using our method. Assuming 100 cases and 100 controls shared between base and target studies, we compared the corrected PRS statistics estimated using our method with the real statistics of individual level PRS obtained using PRSice2. Comparison was carried out under various levels of stratification between base and target population ($F_{st} = 0$, 0.05, and 0.1) and p-value thresholds (denoted by P-thres in the table) for SNP selection. For both methods, mean PRS represents the estimated group mean PRS for cases and controls; and p-val are the t-test p-values comparing the resulting PRS distribution in cases and controls. For PRSice2, we computed these statistics for all the samples in the target population, including the samples shared with the base population (denoted by All samples), as well as only for samples that are present exclusively in the target population (denoted by Non-overlapping Samples).

Fst	P-thres	trait	Our method (`ReACt`) Corrected statistics		`PRSice2` All samples		Non-overlapping Samples	
			mean PRS	p-val	mean PRS	p-val	mean PRS	p-val
0[a]	0.05	cases	0.0003	4.07E-05	0.0012	1.09E-54	0.0003	3.59E-07
		controls	0.0000		-0.0009		0.0000	
	0.005	cases	0.0034	1.28E-04	0.0050	6.02E-39	0.0034	1.20E-04
		controls	0.0024		0.0008		0.0025	
	$5 \cdot 10^{-4}$	cases	-0.0030	2.44E-01	-0.0008	8.96E-12	-0.0028	1.47E-01
		controls	-0.0041		-0.0063		-0.0040	
	$5 \cdot 10^{-5}$	cases	0.0441	7.52E-01	0.0471	2.31E-02	0.0449	5.46E-01
		controls	0.0450		0.0419		0.0464	
0.05[b]	0.05	cases	0.0000	5.57E-54	0.0002	3.55E-111	0.0001	8.64E-88
		controls	-0.0005		-0.0007		-0.0006	
	0.005	cases	0.0001	4.21E-62	0.0001	5.56E-110	0.0000	3.30E-91
		controls	-0.0019		-0.0025		-0.0024	
	$5 \cdot 10^{-4}$	cases	-0.0063	1.51E-50	-0.0067	1.72E-77	-0.0069	3.61E-70
		controls	-0.0112		-0.0124		-0.0124	
	$5 \cdot 10^{-5}$	cases	-0.0234	4.88E-21	-0.0229	3.21E-32	-0.0232	3.04E-29
		controls	-0.0298		-0.0304		-0.0305	
0.1[c]	0.05	cases	0.0001	7.32E-35	0.0004	8.05E-90	0.0004	7.52E-68
		controls	-0.0003		-0.0004		-0.0003	
	0.005	cases	0.0004	2.14E-52	0.0007	8.82E-98	0.0006	3.03E-79
		controls	-0.0014		-0.0017		-0.0015	
	$5 \cdot 10^{-4}$	cases	-0.0048	3.74E-41	-0.0048	6.51E-60	-0.0047	1.32E-52
		controls	-0.0091		-0.0100		-0.0096	
	$5 \cdot 10^{-5}$	cases	0.0109	6.04E-15	0.0087	7.62E-22	0.0088	2.47E-19
		controls	0.0054		0.0021		0.0025	

[a] tested with 550 cases and 550 controls from base and target studies respectively

[b] tested with 1,200 cases and 1,200 controls from base and target studies respectively

[c] tested with 1,200 cases and 1,200 controls from base and target studies respectively

Table S6. Using ReACt to run cc-GWAS cross eight neuropsychiatric disorders. We applied our method for cc-GWAS to the summary statistics of eight neuropsychiatric disorders from PGC. Each spreadsheet reports the genome-wide significant trait differential regions for a pair of disorders analyzed. For each genomic region, statistics and annotation for the leading SNP are reported.

***Excel table.**

5.2 Solving the non-linear system of equations of Section 2.1

For notational simplicity, let $a = a_{i\ell}^{\text{cse}}$, $b = u_{i\ell}^{\text{cse}}$, $c = a_{i\ell}^{\text{cnt}}$, and $d = u_{i\ell}^{\text{cnt}}$. We rewrite eqns. (1)-(4) as

$$\frac{1}{a}+\frac{1}{b}+\frac{1}{c}+\frac{1}{d} = w, \text{ with } w = SE_{i\ell}^2, \tag{18}$$

$$a+b = x, \text{ with } x = 2N_\ell^{\text{cse}}, \tag{19}$$

$$c+d = y, \text{ with } y = 2N_\ell^{\text{cnt}}, \text{ and} \tag{20}$$

$$\frac{a\cdot d}{c\cdot b} = z, \text{ with } z = OR_{i\ell}. \tag{21}$$

Our goal is compute values for the four unknowns a, b, c, and d. Combining eqns. (19) and (20), we get

$$a = x-b, \text{ and} \tag{22}$$

$$c = y-d. \tag{23}$$

Substituting eqn. (22) and eqn. (23) into eqn. (21), we get $(x-b)d = zb(y-d)$, which can be rewritten as

$$b = \frac{xd}{yz-zd+d}. \tag{24}$$

Substituting eqn. (24) into eqn. (22), we get

$$a = x - \frac{xd}{yz-zd+d} = \frac{xyz-xzd}{yz-zd+d}. \tag{25}$$

We now note that all four unknowns can be written as functions of d and other known quantities. Substituting eqn. (23), eqn. (24), and eqn. (25) into eqn. (18), we get

$$\frac{1}{\frac{xyz-xzd}{yz-zd+d}}+\frac{1}{\frac{xd}{yz-zd+d}}+\frac{1}{y-d}+\frac{1}{d} = w.$$

Simplifying the above equation, we get

$$\frac{yz-zd+d}{xz(y-d)}+\frac{yz-zd+d}{xd}+\frac{1}{y-d}+\frac{1}{d} = w,$$

which can be further simplified to

$$(wxz+(1-z)^2)\cdot d^2+(2yz(1-z)-wxyz)\cdot d+(yz(x+yz)) = 0. \tag{26}$$

Eqn. (26) is a quadratic equation on d; its real roots (if they exist) are

$$\{d_1,d_2\} = \frac{-(2yz(1-z)-wxyz)\pm\sqrt{(2yz(1-z)-wxyz)^2-4(wxz+(1-z)^2)(yz(x+yz))}}{2(wxz+(1-z)^2)}.$$

Given d, we can immediately compute a, b, and c using eqns. (23), (24), and (25). In order to determine whether d is equal to d_1 or d_2, we first check whether d_1 or d_2 guarantee that a, b, c, and d are all positive numbers. If both d_1 and d_2 satisfy this constraint, then we choose the *largest* of the two roots, as it solves the following trivial minimization problem:

$$\min_{d\in\{d_1,d_2\}} \frac{a+c}{a+b+c+d}.$$

The above choice is based on the assumption that in summary statistics A_1 (whose frequency is equal to the above fraction) typically denotes the effective (minor) allele. Additionally, our code performs a sanity check for allele alignment across studies given the solution d_1 or d_2.

For the sake of completeness, we also prove that it is not possible for both d_1 and d_2 to be negative. First, note that

$$d_1+d_2 = -\frac{2yz(1-z)-wxyz}{wxz+(1-z)^2} = \frac{yz}{wxz+(1-z)^2}\cdot(wx-2+2z). \tag{27}$$

Using $x = a + b > 0$ and $w = \frac{1}{a} + \frac{1}{b} + \frac{1}{c} + \frac{1}{d} > \frac{1}{a} + \frac{1}{b} > 0$, we get

$$wx > (a+b) \cdot (\frac{1}{a} + \frac{1}{b}) = \frac{(a+b)^2}{ab} = \frac{a^2 + 2ab + b^2}{ab} > 2, \tag{28}$$

which implies that $wx - 2 + 2z > 0$. Combining with eqn. (27), we conclude that $d_1 + d + 2$ is non-negative; recall that w, x, y, and z are all non-negative. Additionally,

$$d_1 \cdot d_2 = \frac{yz(x+yz)}{wxz + (1-z)^2} > 0,$$

which implies that d_1 and d_2 must have the same sign, and since their sum is non-negative, they must both be positive. It is a simple exercise to prove that as long as root(s) exist, at least one of them will guarantee that all values for a, b and c will be positive.

One important exception arises when the discriminant in eqn. (26) is negative. In that case, no real roots exist for the quadratic equation. We do note that, theoretically, this should never happen, since the underlying unknown quantities are positive real numbers. However, stratification correction and genotype missingness could force the discriminant to fall below zero. To address this issue, we inflate w (i.e., the square of the standard error for the respective SNP) and recompute the discriminant. More specifically, we iteratively multiply w by 1.001 (a 0.1% inflation) until a non-negative discriminant is obtained or until 50 iterations are reached. The maximum inflation we allow (after the full 50 iterations) is $1.001^{50} - 1 \approx 5\%$. If after 50 iterations we have failed to find a non-negative discriminant we omit this particular SNP from further analyses. Empirically, for most input SNPs, a real root can be found after at most ten iterations.

5.3 Correction for sample overlap between the base/target studies for group PRS

The existence of shared samples in base (discovery) and target populations can lead to inflation in association between PRS and the trait of interest in the target population [19**?**]. In our case, such overlap will cause higher levels of significance in the t-test comparing the case and control PRS distribution. So far, for conventional PRS, the most widely accepted approach to address this problem is simply to identify the overlapping individuals and remove them from the target population. However, in practice, this is not always possible since it usually requires additional access to the individual level data of the base population. In this section, we introduce a correction for sample overlap between the base and target populations implemented in ReACt that could alleviate such issues.

In the following, we will use the case group as an example. Assume that the sample size for cases of the target population is $N^{\mathtt{cse}}_{\mathtt{target}}$, out of which $N^{\mathtt{cse}}_{\mathtt{shr}}$ are also cases in the base population (overlap). If the probability of each sample being shared between the base and target studies is uniformly distributed in both base and target studies, we would expect the observed mean PRS in target cases $\mathrm{PRS}^{\mathtt{cse}}_{\mathtt{obs}}$ to be a weighted sum of the mean PRS in base cases $\mathrm{PRS}^{\mathtt{cse}}_{\mathtt{base}}$ and the mean PRS of cases that only exist in the target population $\mathrm{PRS}^{\mathtt{cse}}_{\mathtt{target}}$ as follows:

$$\mathrm{PRS}^{\mathtt{cse}}_{\mathtt{obs}} = \frac{N^{\mathtt{cse}}_{\mathtt{shr}}}{N^{\mathtt{cse}}_{\mathtt{target}}} \cdot \mathrm{PRS}^{\mathtt{cse}}_{\mathtt{base}} + \left(1 - \frac{N^{\mathtt{cse}}_{\mathtt{shr}}}{N^{\mathtt{cse}}}\right) \cdot \mathrm{PRS}^{\mathtt{cse}}_{\mathtt{target}}.$$

Therefore, the mean PRS for cases only in the target population is:

$$\mathrm{PRS}^{\mathtt{cse}}_{\mathtt{target}} = \left(\mathrm{PRS}^{\mathtt{cse}}_{\mathtt{obs}} - \frac{N^{\mathtt{cse}}_{\mathtt{shr}}}{N^{\mathtt{cse}}_{\mathtt{target}}} \mathrm{PRS}^{\mathtt{cse}}_{\mathtt{base}}\right) \cdot \frac{N^{\mathtt{cse}}_{\mathtt{target}}}{N^{\mathtt{cse}}_{\mathtt{target}} - N^{\mathtt{cse}}_{\mathtt{shr}}},$$

where $\mathrm{PRS}^{\mathtt{cse}}_{\mathtt{obs}}$ is the uncorrected group mean computed as described in Section 4.3.2. $\mathrm{PRS}^{\mathtt{cse}}_{\mathtt{base}}$ can be obtained by simply setting the target population to be the same as the base population, using base summary statistics to compute group PRS for the target population. Similarly, we can adjust the variance computation as follows:

$$\mathrm{Var}(\mathrm{PRS}^{\mathtt{cse}}_{\mathtt{obs}}) = \left(\frac{N^{\mathtt{cse}}_{\mathtt{shr}}}{N^{\mathtt{cse}}_{\mathtt{target}}}\right)^2 \cdot \mathrm{Var}(\mathrm{PRS}^{\mathtt{cse}}_{\mathtt{base}}) + \left(1 - \frac{N^{\mathtt{cse}}_{\mathtt{shr}}}{N^{\mathtt{cse}}_{\mathtt{target}}}\right)^2 \cdot \mathrm{Var}(\mathrm{PRS}^{\mathtt{cse}}_{\mathtt{target}}). \tag{29}$$

Therefore, the corrected variance will be

$$\mathrm{Var}(\mathrm{PRS}^{\mathtt{cse}}_{\mathtt{target}}) = \left(\mathrm{Var}(\mathrm{PRS}^{\mathtt{cse}}_{\mathtt{obs}}) - \left(\frac{N^{\mathtt{cse}}_{\mathtt{shr}}}{N^{\mathtt{cse}}_{\mathtt{target}}}\right)^2 \cdot \mathrm{Var}(\mathrm{PRS}^{\mathtt{cse}}_{\mathtt{base}})\right) \cdot \left(\frac{N^{\mathtt{cse}}_{\mathtt{target}}}{N^{\mathtt{cse}}_{\mathtt{target}} - N^{\mathtt{cse}}_{\mathtt{shr}}}\right)^2 \tag{30}$$

Similarly,

$$\mathrm{PRS}^{\mathtt{cnt}}_{\mathtt{target}} = \left(\mathrm{PRS}^{\mathtt{cnt}}_{\mathtt{obs}} - \frac{N^{\mathtt{cnt}}_{\mathtt{shr}}}{N^{\mathtt{cnt}}_{\mathtt{target}}}\mathrm{PRS}^{\mathtt{cnt}}_{\mathtt{base}}\right) \cdot \frac{N^{\mathtt{cnt}}_{\mathtt{target}}}{N^{\mathtt{cnt}}_{\mathtt{target}} - N^{\mathtt{cnt}}_{\mathtt{shr}}} \tag{31}$$

and

$$\mathrm{Var}(\mathrm{PRS}^{\mathtt{cnt}}_{\mathtt{target}}) = \left(\mathrm{Var}(\mathrm{PRS}^{\mathtt{cnt}}_{\mathtt{obs}}) - \left(\frac{N^{\mathtt{cnt}}_{\mathtt{shr}}}{N^{\mathtt{cnt}}_{\mathtt{target}}}\right)^2 \cdot \mathrm{Var}(\mathrm{PRS}^{\mathtt{cnt}}_{\mathtt{base}})\right) \cdot \left(\frac{N^{\mathtt{cnt}}_{\mathtt{target}}}{N^{\mathtt{cnt}}_{\mathtt{target}} - N^{\mathtt{cnt}}_{\mathtt{shr}}}\right)^2 \tag{32}$$

for controls. Then, the corrected p-value will be based on a t-test using the corrected mean and variance and an adjusted degree of freedom:

$$df_{\mathtt{target}} = N^{\mathtt{cnt}}_{\mathtt{target}} + N^{\mathtt{cse}}_{\mathtt{target}} - (N^{\mathtt{cnt}}_{\mathtt{shr}} + N^{\mathtt{cse}}_{\mathtt{shr}}) - 2.$$

This is a straightforward correction on the target PRS using the scores of the base population that one would use if there were no stratification between the base and target populations. In practice, this idealized scenario does not hold. In order to deal with the stratification between the base and target populations, prior to any correction, we shift the scores for base cases and controls by aligning the base population PRS means to the target population as follows:

$$\mathrm{PRS}^{\mathtt{cse}\star}_{\mathtt{base}} = \mathrm{PRS}^{\mathtt{cse}}_{\mathtt{base}} - (\mathrm{PRS}_{\mathtt{base}} - \mathrm{PRS}_{\mathtt{target}}),$$
$$\mathrm{PRS}^{\mathtt{cnt}\star}_{\mathtt{base}} = \mathrm{PRS}^{\mathtt{cnt}}_{\mathtt{base}} - (\mathrm{PRS}_{\mathtt{base}} - \mathrm{PRS}_{\mathtt{target}}).$$

In the above, $\mathrm{PRS}_{\mathtt{base}}$ and $\mathrm{PRS}_{\mathtt{target}}$ are mean PRS for the base and target populations respectively:

$$\mathrm{PRS}_{\mathtt{base}} = \frac{N^{\mathtt{cnt}}_{\mathtt{base}} \cdot \mathrm{PRS}^{\mathtt{cnt}}_{\mathtt{base}} + N^{\mathtt{cnt}}_{\mathtt{base}} \cdot \mathrm{PRS}^{\mathtt{cnt}}_{\mathtt{base}}}{N^{\mathtt{cse}}_{\mathtt{base}} + N^{\mathtt{cnt}}_{\mathtt{base}}},$$
$$\mathrm{PRS}_{\mathtt{target}} = \frac{N^{\mathtt{cnt}}_{\mathtt{target}} \cdot \mathrm{PRS}^{\mathtt{cnt}}_{\mathtt{target}} + N^{\mathtt{cnt}}_{\mathtt{target}} \cdot \mathrm{PRS}^{\mathtt{cnt}}_{\mathtt{target}}}{N^{\mathtt{cse}}_{\mathtt{target}} + N^{\mathtt{cnt}}_{\mathtt{target}}}.$$

In practice, we use $\mathrm{PRS}^{\mathtt{cse}\star}_{\mathtt{base}}$ and $\mathrm{PRS}^{\mathtt{cnt}\star}_{\mathtt{base}}$ instead of $\mathrm{PRS}^{\mathtt{cse}}_{\mathtt{base}}$ and $\mathrm{PRS}^{\mathtt{cnt}}_{\mathtt{base}}$ in equations (29)-(32) for correction. We evaluated the performance of this correction scheme by introducing sample overlaps between the base and target populations using the same simulation model as the one we used to evaluate the performance of our group PRS approach. We computed the real individual level PRS using PRSice2, from which we obtained the inflated PRS descriptive statistics (group mean, standard deviation, and t-test p-value) for all target samples, including the ones that are shared with the base population. We also computed PRS statistics for samples that are present only in the target population as the ground truth. We compared results from our corrected group PRS method to the PRS statistics for the samples that are exclusive to the target population computed using PRSice2. Results on synthetic data demonstrated that our correction can drastically alleviate the inflation in p-values that is the result of sample overlap the between base and target populations. See Table S5, which shows representative results from our experimental evaluations. If the number of overlapping samples is unknown to the user, we apply the approach proposed in [28] to get an estimate of the overlapping sample size and we correct the output statistics accordingly. Note that this correction approach is based on the assumption that all samples having an equal probability of being shared between the base and target populations, which might be unrealistic in certain settings.

5.4 Speeding up the logistic regression computation

Recall that in section 4.2.1, for any SNP i, if we try to formulate the computation of elements in $\boldsymbol{H}$ and $\boldsymbol{G}$ in one iteration, they will be:

$$\boldsymbol{H}_{uv} = \sum_{j=1}^{N} d_j \cdot \boldsymbol{X}_{ju}\boldsymbol{X}_{jv} \tag{33}$$

and

$$\boldsymbol{G}_u = \sum_{j=1}^{N} d_j \cdot z_j \cdot \boldsymbol{X}_{ju} \tag{34}$$

where $u, v \in \{0,1,2\}$.

Same as in section 4.2.1 , we dropped the subscript i from d_j, z_j and $\boldsymbol{X}$. If we follow these equations, when the sample size

$\sum_{\ell=1}^{L} N_\ell$ increases, the computational burden will increase linearly. However, in practice this step can be achieved with an $O(L)$ complexity, as long as we take advantage of the fact that all elements of $\boldsymbol{X}$ are discrete values involving only 0, 1, 2 and study indicators $I_{i\ell}$. This indicates that both $d_j \cdot \boldsymbol{X}_{ju}\boldsymbol{X}_{jv}$ and $d_j \cdot z_j \cdot \boldsymbol{X}_{ju}$ can only take a few possible values. In fact, since there are only $3 \cdot L$ possibilities for $\boldsymbol{X}_{j*}$ (3 different genotypes $\cdot$ L different studies), there are also only 6 possible values for d_j. We denote them as $d_{\ell\mathtt{n}}$, with $\ell \in \{1,\dots,L\}$ and $\mathtt{n} \in \{0,1,2\}$. Therefore, as an example, $d_{\mathtt{10}}$ will be the value of d_j for a sample j if it belongs to study 1 and has a genotype of A2A2. Similarly, for z_j, since y_j is involved in this computation, we need to consider in total $3L \cdot 2 = 6L$ possible values as $\boldsymbol{y}$ is binary indicator for the phenotypes. We denote those $6 \cdot L$ possible values as $z_{\ell\mathtt{n}}^{\mathtt{cse}}$ for cases and $z_{\ell\mathtt{n}}^{\mathtt{cnt}}$ for controls respectively. Then $z_{\mathtt{10}}^{\mathtt{cnt}}$ will represent the value of z_j for a sample j if it belongs to study 1, has a genotype of A2A2 and meanwhile is a control. Then we only need to plug in the element of $\boldsymbol{X}$ based on the u, v values of interest.

Noticing this, if we just count the occurrence of those values, the summation can be found out easily. This can be done using the genotype counts that we have already computed in section 4.1.3 . Therefore, for any SNP i with occurrence $N_{i\ell}^{\text{cnt}}(n)$ of each genotype n and indicator $I_{i\ell}$ for each input study, in an iteration of the IRLS, we can compute all $d_{\ell\mathtt{n}}$ and $z_{\ell\mathtt{n}}^{\mathtt{cnt}}$ needed for this SNP as described in 1 . Then for this iteration, we shall have:

$$\boldsymbol{H}_{00} = \sum_{\ell=1}^{L}\sum_{n=0}^{2} d_{\ell\mathtt{n}} \cdot (N_{i\ell}^{\text{cnt}}(n) + N_{i\ell}^{\text{cse}}(n)) \tag{35}$$

$$\boldsymbol{H}_{01} = \boldsymbol{H}_{10} = \sum_{\ell=1}^{L}\sum_{n=0}^{2} n \cdot d_{\ell\mathtt{n}} \cdot (N_{i\ell}^{\text{cnt}}(n) + N_{i\ell}^{\text{cse}}(n)) \tag{36}$$

$$\boldsymbol{H}_{02} = \boldsymbol{H}_{20} = \sum_{\ell=1}^{L}\sum_{n=0}^{2} I_{i\ell} \cdot d_{\ell\mathtt{n}} \cdot (N_{i\ell}^{\text{cnt}}(n) + N_{i\ell}^{\text{cse}}(n)) \tag{37}$$

$$\boldsymbol{H}_{11} = \sum_{\ell=1}^{L}\sum_{n=0}^{2} n^2 \cdot d_{\ell\mathtt{n}} \cdot (N_{i\ell}^{\text{cnt}}(n) + N_{i\ell}^{\text{cse}}(n)) \tag{38}$$

$$\boldsymbol{H}_{12} = \boldsymbol{H}_{21} = \sum_{\ell=1}^{L}\sum_{n=0}^{2} n \cdot I_{i\ell} \cdot d_{\ell\mathtt{n}} \cdot (N_{i1}^{\text{cnt}}(n) + N_{i1}^{\text{cse}}(n)) \tag{39}$$

$$\boldsymbol{H}_{22} = \sum_{\ell=1}^{L}\sum_{n=0}^{2} I_{i\ell}^2 \cdot d_{\ell\mathtt{n}} \cdot (N_{i1}^{\text{cnt}}(n) + N_{i1}^{\text{cse}}(n)) \tag{40}$$

and

$$\boldsymbol{G}_{0} = \sum_{\ell=1}^{L}\sum_{n=0}^{2} d_{\ell\mathtt{n}} \cdot (z_{\ell\mathtt{n}}^{\mathtt{cnt}} \cdot N_{i\ell}^{\text{cnt}}(n) + z_{\ell\mathtt{n}}^{\mathtt{cse}} \cdot N_{i\ell}^{\text{cse}}(n)) \tag{41}$$

$$\boldsymbol{G}_{1} = \sum_{\ell=1}^{L}\sum_{n=0}^{2} n \cdot d_{\ell\mathtt{n}} \cdot (z_{\ell\mathtt{n}}^{\mathtt{cnt}} \cdot N_{i\ell}^{\text{cnt}}(n) + z_{\ell\mathtt{n}}^{\mathtt{cse}} \cdot N_{i\ell}^{\text{cse}}(n)) \tag{42}$$

$$\boldsymbol{G}_{2} = \sum_{\ell=1}^{L}\sum_{n=0}^{2} I_{i\ell} \cdot d_{\ell\mathtt{n}} \cdot (z_{\ell\mathtt{n}}^{\mathtt{cnt}} \cdot N_{i\ell}^{\text{cnt}}(n) + z_{\ell\mathtt{n}}^{\mathtt{cse}} \cdot N_{i\ell}^{\text{cse}}(n)) \tag{43}$$

Eqn. (35)-(43) grant us fast update of $\boldsymbol{w} = \boldsymbol{H}^{-1}\boldsymbol{G}$ in each iteration. We can do this repeatedly in the IRLS until convergence to get the final result $\widetilde{\boldsymbol{w}}$.
